# Self-folding soft-robotic chains with reconfigurable shapes and functionalities

**DOI:** 10.1038/s41467-023-36819-z

**Published:** 2023-03-07

**Authors:** Hongri Gu, Marino Möckli, Claas Ehmke, Minsoo Kim, Matthias Wieland, Simon Moser, Clemens Bechinger, Quentin Boehler, Bradley J. Nelson

**Affiliations:** 1 Institute of Robotics and Intelligent Systems, ETH Zurich, Zurich, Switzerland; 2 Department of Physics, University of Konstanz, Konstanz, Germany

**Keywords:** Engineering, Mechanical engineering, Soft materials

## Abstract

Magnetic continuum soft robots can actively steer their tip under an external magnetic field, enabling them to effectively navigate in complex in vivo environments and perform minimally invasive interventions. However, the geometries and functionalities of these robotic tools are limited by the inner diameter of the supporting catheter as well as the natural orifices and access ports of the human body. Here, we present a class of magnetic soft-robotic chains (MaSoChains) that can self-fold into large assemblies with stable configurations using a combination of elastic and magnetic energies. By pushing and pulling the MaSoChain relative to its catheter sheath, repeated assembly and disassembly with programmable shapes and functions are achieved. MaSoChains are compatible with state-of-the-art magnetic navigation technologies and provide many desirable features and functions that are difficult to realize through existing surgical tools. This strategy can be further customized and implemented for a wide spectrum of tools for minimally invasive interventions.

## Abstract (web summary)

Minimally invasive surgeries call for surgical tools that can work at the mesoscale. Here, Gu et al. present a class of magnetic soft robotic chains that can self fold into large assemblies with stable configurations using a combination of elastic and magnetic energies stored in printed chain material.

## Introduction

By minimizing the incision size through the skin and soft tissues, minimally invasive surgeries (MIS) provide numerous benefits to patients (less pain, faster recovery, fewer infections, etc.) compared to conventional open surgeries^1–3^. The procedures often involve pushing small surgical tools through a narrow channel in a supporting catheter sheath, navigating a tortuous path, and performing surgical operations in constrained in vivo environments. Robotic technologies are transforming almost all aspects of MIS by providing higher precision and stability, high-quality imaging and 3D modeling, automated navigation, teleoperation, precision drug delivery, and potentially fully automated surgical interventions^4,5^. Designing intelligent miniaturized surgical tools is at the forefront of the robotic revolution^4–11^. Recent developments in advanced microfabrication (e.g., multimaterial 3D printing^12^, micromolding^13,14^) and functional soft materials (e.g., shape memory polymers, stimuli-responsive hydrogels) create opportunities for the next generation of MIS tools^15^. Compared to pull-wire, hydraulic, and pneumatic actuation mechanisms, magnetically driven soft robots do not require power to be transmitted through the cable; instead, they are transmitted by a piece of magnetic material on the soft bendable tip. This simplified design favors magnetically driven surgical tools for miniaturization^16–19^, potentially as a platform to integrate multiple functional components (e.g., force sensors, cameras, lasers, drug carriers, etc.) for fully functional and advanced microsurgical tools.

**Fig. 1:**
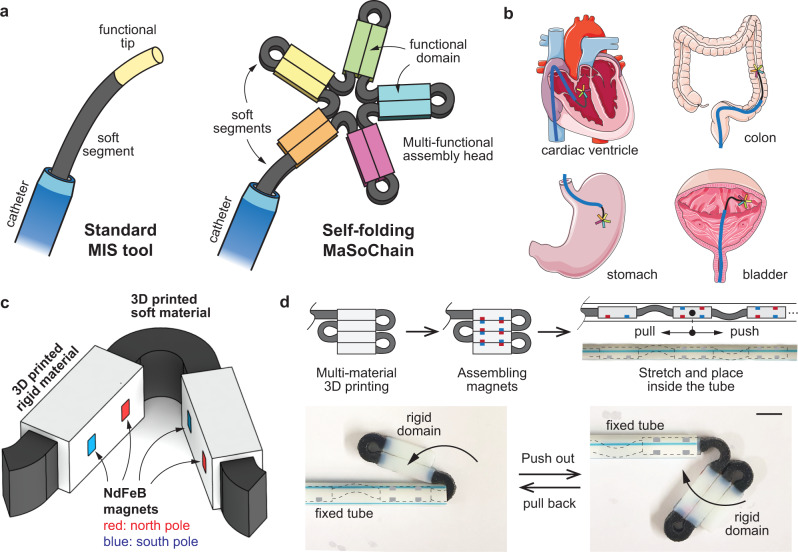
Concept and structure of magnetic soft-robotic chains (MaSoChains). **a** A schematic illustration of a standard MIS tool and a self-folding MaSoChain. The MaSoChains can self-fold into large assemblies at the tip of the catheter with multiple functional domains. **b** Potential application scenarios for MaSoChains. When pushed out of the sheathing catheter (marked in blue), MaSoChain can fold into large functional structures (colored star) in relatively open chambers in the human body (e.g., heart ventricles, colon, stomach, bladder). **c** The basic self-folding unit of MaSoChains, composed of rigid segments (in white) connected by soft segments (in black). Small NdFeB magnets are embedded at the same height as the surrounding surface. **d** (upper) A schematic illustration of the preparation of the MaSoChains. After assembling the small magnets, the MaSoChains are stretched and placed inside the sheathing tube, where elastic and magnetic energies are stored. (lower) The folding process of MaSoChain is initiated when a new segment is pushed out of the sheathing tube. The elastic energy (stored in the soft segment) folds the MaSoChain, and magnetic energy secures a stable assembly (marked as the rigid domain). MaSoChain is disassembled by pulling back with guiding of the fixed tube. The scale bar is 5 mm.

Despite the increasing complexity and functionality of surgical tools, the overall size is still limited by the dimension of the inner lumen of the catheter sheath and the size of the incision ports and natural orifices in the human body. This size limitation prevents large tools and functional structures from entering targeted locations, even if the targeted locations are relatively open (as in Fig. 1b, e.g., the bladder, heart chambers, abdominal cavity, etc.). The need to pass through these small openings poses challenges for the design of surgical tools, particularly for system integration, assembly, and packaging. In some cases, elastic folding can be implemented as a method to overcome the size limitations. For example, precurved catheter tips can assist navigation in vascular networks^20^, and superelastic nitinol stents can expand to a larger diameter when released from the endovascular catheter. However, the shape changes of these devices are limited to very simple geometries and lack a generalized strategy to construct irregular structures. Another limitation associated with the existing surgical tools is that they are usually optimized for a single function (e.g., navigation, gripping). In endovascular surgeries, surgeons often need to change tools multiple times by pulling out and inserting different catheters, guidewires, and other surgical tools^21,22^. Repeated swapping of MIS tools can substantially prolong the procedure, hence increasing the risk of vessel dissection and distal embolism^22^.

Reconfigurable soft robots can potentially overcome these limitations by actively changing their shapes in situ. The shape-changing capability allows the surgical tools to be inserted into the catheter in a narrow configuration, and they can then transform into large functional structures upon reaching the targeted site^23–25^. Furthermore, the shapes can be preprogrammed with different functional segments, allowing the soft robot to quickly switch between different functions, reducing the overall surgery time and risk of infection. However, implementing shape reconfigurability into surgical soft robots is challenging. There are two main types of shape morphing soft robots. One uses rigid links with distributed actuators (electric, pneumatic, hydraulic, etc.) along the soft body and can actively change and hold shapes by precisely tuning the driving signal of the actuators. Such robots have large degrees of freedom in shape changing^26–31^. Unfortunately, the structures cannot be easily miniaturized to relevant scales for MIS (a few millimeters or smaller) without losing actuation power. In addition, the parallel control architecture to access the individual actuator resists further system integration. The other type of shape morphing soft robot uses stimuli-responsive soft materials (hydrogels, liquid crystal elastomers, shape memory alloys and polymers, composite magnetic materials) with carefully designed shapes and structures^32,33^. Under an environmental stimulus (pH, temperature, light, magnetic field, etc.), soft robots can deform into various complex structures^34–36^. The intrinsic softness provides them with superior adaptability to negotiate the complexity in in vivo environments. However, the same feature makes them prone to disturbances, which is unsuitable for performing reliable surgical procedures that require a large force and high accuracy. One potential solution is to implement phase-changing materials to vary the stiffness of the structures^37–39^. However, complex shape reconfiguration has yet to be realized since it requires integration with multiple control modules that are accessible individually. Similar to the first type of shape-morphing soft robot with distributed actuators, it raises significant challenges in miniaturization, integration, and packaging for in vivo applications.

**Fig. 2:**
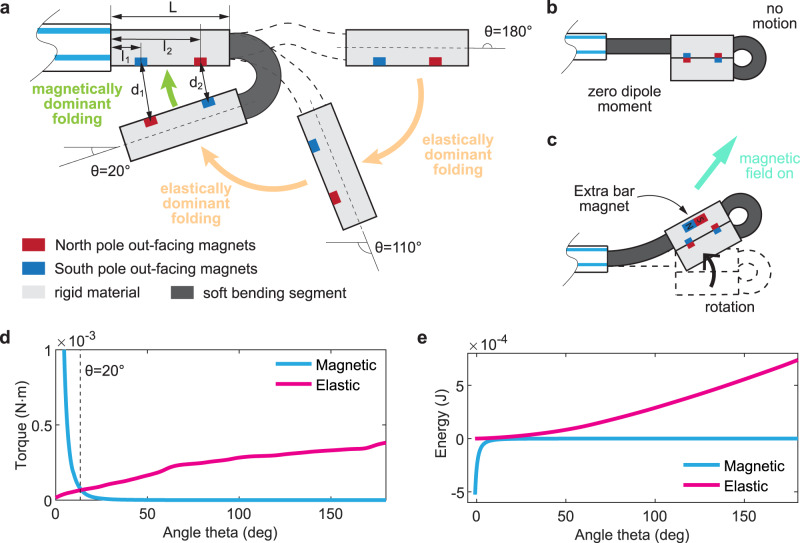
A smooth folding process with a combination of elastic and magnetic energies. **a** An illustration of a typical folding process. The free rigid segment is folded from the initial position (θ = 180°) to the final assembly position (θ = 0°). From the initial position, the folding process is dominated by the elastic torque until it is close to the final assembly position (θ = 20°) where the assembly magnets close. **b**, **c** Demonstration of the zero dipole moment for the assembly magnets that do not bend under an external magnetic field. Bending is made possible by adding an extra bar magnet. **d**, **e** The overall torque and energy associated with the elastic and magnetic interactions at different folding angles θ. The elastic energy is experimentally derived from experimental test 1 in Supplementary Fig. 1. The detailed modeling can be found in Methods: Magnetic interaction in the assembly process.

Here, we introduce a class of magnetic soft-robotic chains (MaSoChains) that can be reconfigured into programmable shapes by pushing them out of a guiding catheter. The MaSoChains are composed of 3D printed soft and rigid segments assembled with NdFeB magnets. When pushed out of the catheter sheath, the prestretched soft elastic segment will start to bend and allow neighboring rigid segments to join together (Fig. 1d), as the NdFeB magnets will attract and lock the assembly shapes (Fig. 2a). This robust folding mechanism can be reversed and reassembled again by pulling and pushing the MaSoChain back and forth along the tube. This shape-changing strategy can be implemented in a wide selection of programmable shapes that are significantly larger than the insertion dimension. This strategy opens up new opportunities in MIS tool design and allows for some unforeseen features, including an enhanced reachable area for the tip of the continuum soft robot and a large gripper. Combined with flexible printed circuit boards (flexible PCBs), we can further expand the functionality of MaSoChain as a platform for integrating both off-the-shelf and customized electronic devices for sensing, actuation, and computation. In the end, we demonstrate a functional MaSoChain-based tethered capsule endoscope with an onboard camera, a steering magnet, and a working channel to perform biopsy.

## Results

### Design and fabrication of magnetic soft-robotic chains (MaSoChains)

The MaSoChains are composed of alternating soft (black) and rigid (white) segments (Fig. 1). We prototyped our samples using multimaterial 3D printing (Objet 360, Stratasys Inc.). This process allows easy integration of soft and rigid materials with relatively complex geometries. Sacrificial supporting materials are covered on all surfaces, resulting in a uniform matt finish on all surfaces. After completely removing all supporting materials, small NdFeB magnets (1.2 × 1.0 × 0.5 mm) are manually assembled into the matching cavity on the rigid segments with instant glue. As shown in Fig. 1c, two pairs of magnets are used to achieve stable magnetic assembly between two rigid objects. The magnets are embedded into the rigid parts with a flat surface so that only the north or south poles are facing outward. The magnetic dipole direction of the assembly magnets is perpendicular to the assembling surface to maximize the assembly force. The contact assembly between two permanent magnets ensures a stable configuration, although it is still possible to separate the magnets through the pushing and pulling motion of the chain robot inside the tube^40^.

We intentionally designed the dipole directions of two pairs of assembly magnets to be opposite (marked red and blue in Fig. 1c) so that the overall dipole moment on each rigid segment is zero. This means that the magnetic torque generated by the assembly magnets will cancel out under a uniform external magnetic field. This design enables the decoupling of two very different purposes (magnetic assembly and magnetic actuation) using NdFeB magnets^41^. One can embed extra NdFeB magnets, other than the NdFeB magnet pairs, to encode the desired dipole moment for programmable bending motion under a dynamic external magnetic field (Fig. 2b, c)^41^. The MaSoChains are printed in the same shape as the final assembly (Fig. 1d), with a small gap (0.3 mm) between the rigid segments to prevent the parts from being glued together during printing. The MaSoChains are then cleaned, assembled with magnets, stretched, and placed inside the tube. When pushed out, the elastic energy stored in the soft segment is released, and the structure folds into its original shape, which is then locked by the assembly magnets into the final position. This process can be reversed by simply pulling the MaSoChain back into the tube, which will disassemble the magnet pairs and deform the soft segment.

### A combination of elastic and magnetic energies

Successful folding relies on both magnetic and elastic energies that are stored when stretched and placed inside the base tube. As shown in Fig. 2, when a segment is pushed out of the tube (*θ* = 0°), the elastic torque is large and quickly bends the free segment toward the final folded position. With an increasing bending angle *θ*, the elastic torque decreases and eventually becomes zero (at *θ* = 180°), and the MaSoChain is assembled to the targeted assembly (same as printed geometries). To secure robust assembly when the elastic torque is weak, we implemented two pairs of assembly magnets with touching surfaces (Fig. 1c). Due to the strong dipole‒dipole interaction between two NdFeB magnets, the magnetic interaction dominates the folding process at large *θ* angles. The elastically dominant folding regime and the magnetically dominant folding regime are marked in Fig. 2a.

By tuning the relative strength of elastic and magnetic interactions, a smooth folding process is achieved (Supplementary Movies 1 and 2). To understand the elastic torque generated from the soft segments at different bending angles, we experimentally studied the force‒displacement curve using a slowly rotating magnetic field (Supplementary Fig. 1d). The results show that the 3D-printed soft material is hyperelastic (Supplementary Fig. 1c) with a characteristic nonlinear response to the load (Fig. 2c). We modeled the magnet interaction through magnetic dipole‒dipole interactions between the assembly pairs (see Methods), and the results are shown in Fig. 2c, d. One can fine-tune the relative strength of the magnetic interaction by changing the size and geometry of the assembly magnets, as the magnetic dipole‒dipole interaction is short-ranged and quickly decays with a relative distance of ~*r*^(−5)^41^. Similarly, 3D-printed soft segments can be designed with various geometries to match the ideal elasticity. The soft material that is available for our 3D printer is Agilus (tensile strength: 2.4–3.1 MPa, maximum elongation before the break: 220–270%). One could use other high-performance elastic materials (including silicone elastomers and superelastic nitinol), but then the simple integration of multimaterial 3D printing would not be possible. As a result, the combination of elastic and magnetic energies (~5^(−4) J) will ensure a minimal folding force to assemble at all angles, which can overcome potential unknown disturbances in complex in vivo environments.

### Folding into functional structures

**Fig. 3:**
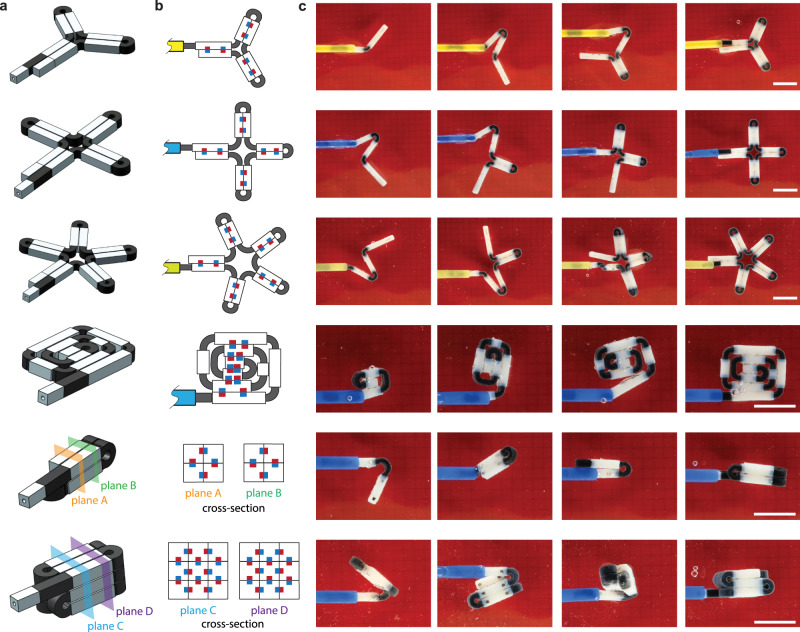
Dynamic folding process of complex 2D and 3D geometries. The 3D design, structure diagram, and dynamic folding process are shown in panels **a**, **b**, and **c**, respectively. The scale bar is 2 cm. The complete folding and unfolding process can be seen in Supplementary Movies 1 and 2.

The capability for self-folding into a variety of geometries opens up many possibilities for MaSoChains. We demonstrated the complete assembly and disassembly processes of some representative 2D and 3D geometries, as shown in Fig. 3 and Supplementary Movies 1 and 2. Due to the combination of magnetic and elastic energy, the folding process is highly repeatable among all geometries and can be controlled by the insertion length of the sheath. To show the potential impact of this technology in the field of MIS, we implemented this strategy in the following two examples, demonstrating interesting features that would be very challenging for conventional MIS tools.

**Fig. 4:**
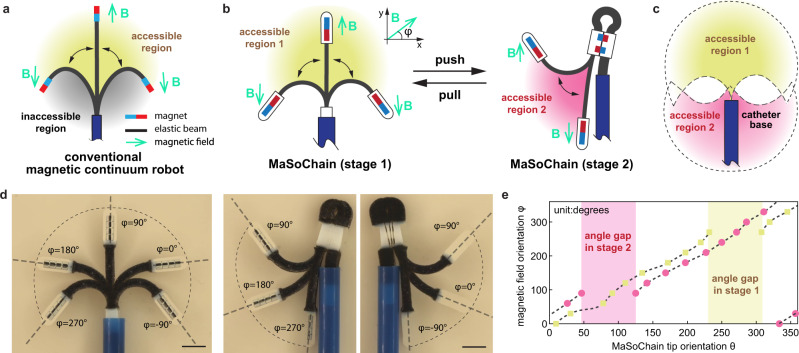
Expanded accessible regions for magnetically guided catheters. **a** A schematic illustration of a conventional magnetic continuum robot. Based on the reachable angles of the tip under a dynamic magnetic field, we marked the surroundings as “accessible region” (in yellow) and “inaccessible region” (in black). **b** Accessible regions of two stages of the MaSoChain by pushing and pulling relative to the sheathing tube/catheter. **c** The experimental results of overlapped images of the catheter position, orientation (dashed straight line), and corresponding magnetic field orientation φ. The external magnetic field is 10 mT rotating inside the same plane. The complete demonstration can be seen in Supplementary Movie 3. **d** The complete accessible regions (yellow and red) of both working stages of the MaSoChain, referring to the base tube/catheter. The scale bar is 5 mm. **e** Experimental comparison between the tip angle and the magnetic field angle. Under a 360-degree magnetic field, each stage has a gap that cannot be covered; however, by combining the two stages, a full angle of 360° is covered and provides more options for two different configurations with the same contact angle.

In previously reported magnetic continuum robots, the tip is only able to reach a limited accessible region (marked in Fig. 4a) and is dependent on the elasticity and the external magnetic field. However, the basal regions are always difficult to reach and are marked as an “inaccessible region” in Fig. 4a. When approached, the catheter can abruptly snap through to the other side due to its intrinsic instability^42,43^. This feature poses challenges in some surgical procedures where access to all regions is needed. For example, in cardiac ablations, a catheter is steered to target sites within the heart chambers to deliver radiofrequency energy and ablate the tissues responsible for cardiac arrhythmias^44^. The treatment of atrial flutter, one of the most common types of cardiac arrhythmias, consists of accessing the right atrium from the inferior *vena cava* (IVC) and performing a linear lesion from the base of the IVC to the tricuspid ring, which is particularly challenging because the instrument must adopt a retrograde shape^44^. To resolve this challenge, we implemented a folding MaSoChain that can access all angles without compromising the dynamic stability. As shown in Fig. 4b, the accessible region of the catheter tip is similar to that of a conventional magnetic continuum robot. However, by pushing the MaSoChain out with an additional segment, the MaSoChain folds 180 degrees backward to face the direction where the catheter is inserted. By combining the accessible regions of both stages, the tip of the MaSoChain can access all 360 degrees in the demonstrated 2D plane (Fig. 4d).

**Fig. 5:**
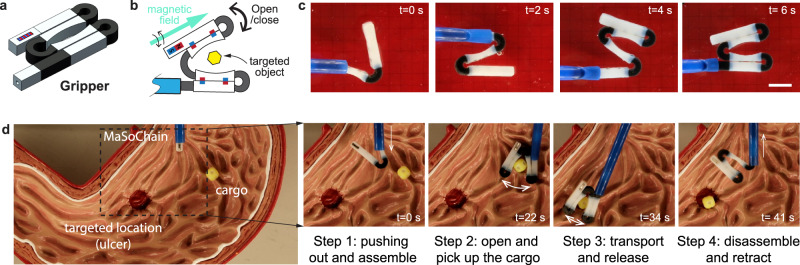
Large magnetic tweezers from self-folding MaSoChain. **a**, **b** The design and structure diagram of a large magnetic tweezer using a self-folding MaSoChain. **c** The folding process of the magnetic tweezer. The complete folding process can be seen in Supplementary Movie 4. The scale bar is 1 cm. **d** Demonstration of targeted cargo transport in a plastic stomach model. The demonstration is composed of four steps (see panel **d**), and the gripping motion is actuated by manually moving a large NdFeB magnet (see Methods). The full demonstration can be seen in Supplementary Movie 4.

In another example, we demonstrate the capability of MaSoChain to fold into a tweezer that is much larger than the diameter of the sheathing tube. As shown in Fig. 5, the gripper is composed of two rigid domains connected by the soft 3D-printed elastic material. Each rigid domain is assembled through two rigid segments and locked with assembly magnets. To achieve the gripping motion, we embed a large dipole magnet next to the zero-dipole assembly magnet pairs. As a result, the two folded arms can grip objects in response to an external magnetic field. We have demonstrated complete assembly, grip, transport, release, and disassembly processes in a stomach model (Fig. 5d and Supplementary Movie 4).

### Integration with flexible PCBs

We are currently witnessing a trend in combining flexible electronics with MIS tools^45–48^. Electronic components can be seamlessly integrated into small surgical tools for sensing^45^, drug delivery^46^, and endothelial sealing^48^. Flexible electronics can function as a core platform to integrate electrically controlled functional components toward increasingly intelligent MIS tools that combine sensing, actuating, and local computing power^49,50^.

Here, we integrate commercially available flexible PCBs with MaSoChains and demonstrate some promising features enabled by self-folding into programmable geometries. We used dual-layer flexible PCBs (based on polyimide) with a thickness of 116 µm (see Methods: Design of flexible electronics). To compensate for the stiffness increase from the flexible electronics and ensure a smooth folding process, we increased the dimensions of the overall MaSoChain (cross-section: 5 × 5 mm, see Methods: 3D printed composite MaSoChains). We implemented round rigid pins to allow the flexible PCB to pass through the 3D-printed soft segments without having to break them into two sections (Supplementary Fig. 2).

**Fig. 6:**
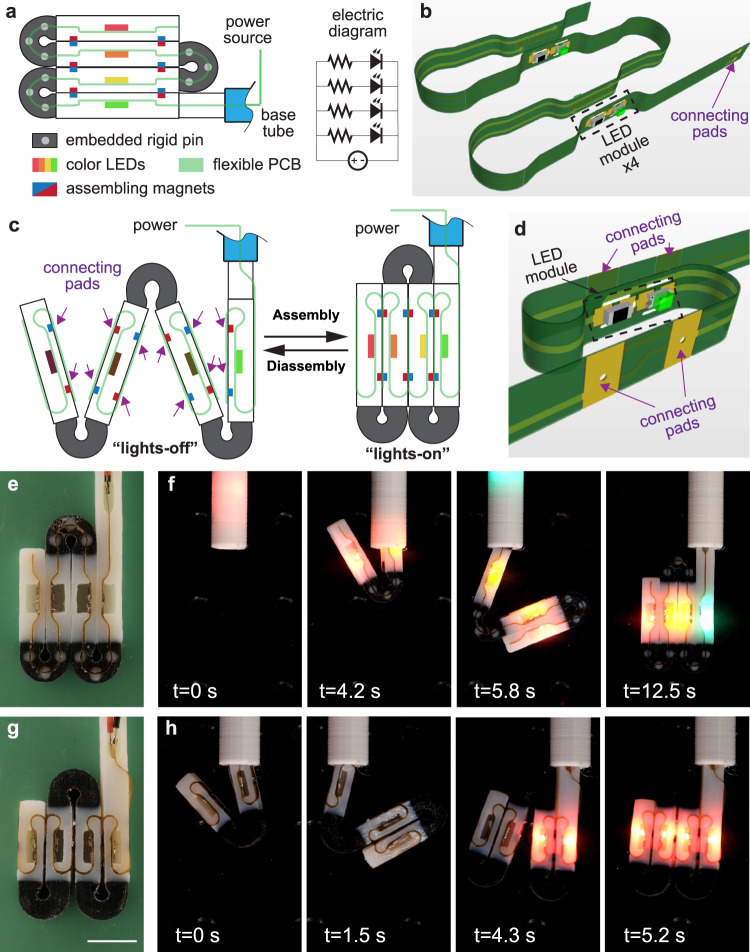
Integration of flexible PCBs and LEDs into the functional MaSoChains. **a** An illustration of the structure and electrical connections of flexible electronics. **b** A bird’s eye view of a completely folded flexible PCB assembled with LEDs. **c** Illustration of shape-sensing MaSoChains through local electrically connecting pads (marked with purple arrows) that will only light up when the MaSoChain is correctly assembled. **d** Bird’s eye view of the flexible PCB on a rigid segment with one LED in the middle. **e**, **f** Optical image and folding process of MaSoChain integrated with colored LEDs on a flexible PCB. **g**, **h** Optical image and folding process of a shape-sensing MaSoChain equipped with a separated flexible PCB with colored LEDs. The LED will turn off if the connecting pads are not correctly assembled. The scale bar is 10 mm.

**Fig. 7:**
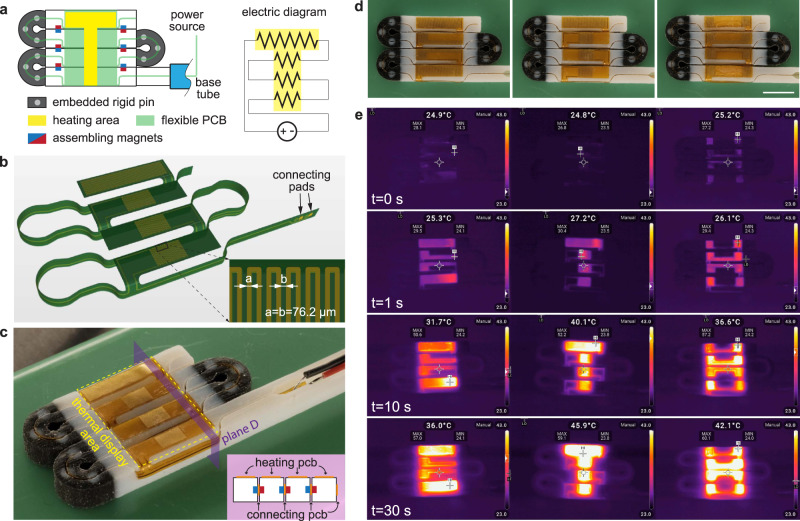
Programmable heating surface through self-folding MaSoChains. **a** An illustration of the mechanical structure and electrical configurations between heat elements on the flexible PCB. **b** An illustrated bird’s eye view of a 3D-structured flexible PCB geometry after assembly. **c** Optical image of the sample after assembly with a thermal display area (marked by dashed yellow lines). A cross-section view (the purple rectangle) shows the relative position between the assembly magnets and the folded flexible PCB. **d**, **e** The visible and time-lapse infrared images of the letters “ETH” on the folded structure, which heats up over time from room temperature. The detailed experimental conditions can be found in Methods. The scale bar is 10 mm.

In Fig. 6, we show a string of colored LEDs integrated into zigzag folded MaSoChains. The LEDs are on a single strip of flexible PCB that is folded and embedded into a MaSoChain (Supplementary Movie 5). The power is connected through the two metal pads (Fig. 6b and Fig. 7b, “connecting pads”) at the end of the flexible PCB. We also demonstrated a shape sensing structure where the LEDs light up only when they are successfully assembled. To achieve this, we designed short PCB strips that are separately integrated into the individual rigid segments of the MaSoChain (Fig. 6c, d). On the assembly surface, two pairs of connecting metal pads cover the assembly magnets. When the structure is assembled, the assembly magnets secure a robust connection between the connecting pads of neighboring flexible PCB segments to conduct electricity. As a result, light is an indicator of the current state of the geometry of the MaSoChain assembly (Fig. 6h and Supplementary Movie 6).

In a separate demonstration, we showed how MaSoChain with integrated flexible electronics can be folded into a large heating surface with preprogrammed thermal patterns. As shown in Fig. 7 and Supplementary Movie 7, we designed the heating surface through thin zigzag copper trace patterns on a selected area that can fold into a large surface on top of the assembly. Flexible PCBs are designed to fold into a 3D geometry by conforming to the surface of the MaSoChains (Supplementary Fig. 3). A thermal pattern emerges when the MaSoChain is connected with external power, and a stabilized temperature of approximately 45 degrees Celsius is reached after 10 s.

### Demonstration of a self-folding tethered capsule endoscope

**Fig. 8:**
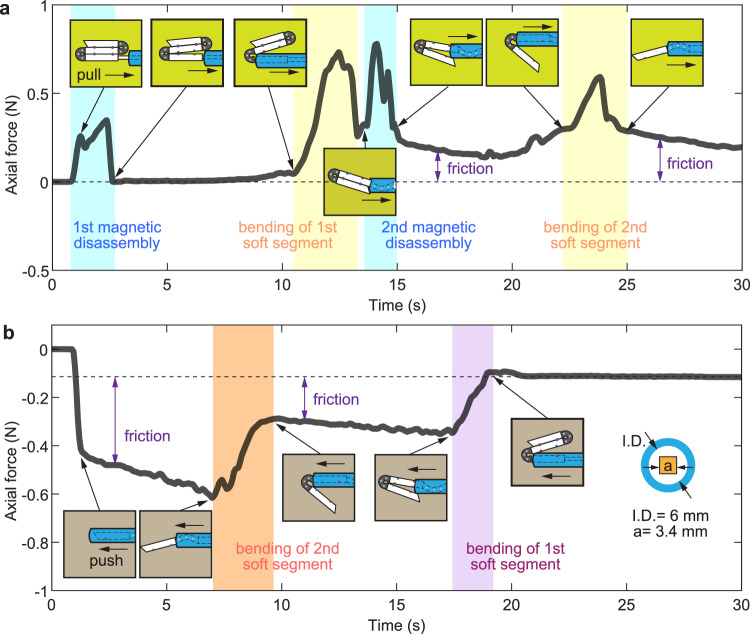
Force characterization of MaSoChains assembly and disassembly inside a medical grade thoracic catheter. The pulling/pushing force along the axial direction during the disassembly (panel **a**) and assembly (panel **b**) are measured. The pushing and pulling speed is 5 mm per second. The measurement rate is 1 kHz, but the curve is smoothed with a moving average of the nearest 200 points. The folding process involved bending of the soft segments and magnetic disassembly. Key moments are marked along the force‒displacement curve with small inset diagrams to show the instant shapes of MaSoChains. More information can be seen in Supplementary Movie 8 and the supplementary information.

The reliable operation of MaSoChain requires mechanical interaction with the sheathing tube during the assembly and disassembly process. To understand this process, we experimentally investigated the reaction force in the axial direction on the MaSoChain when pushing and pulling out of a medical grade PVC thoracic catheter. The force dynamically changes during the disassembly process, as shown in Fig. 8a and Supplementary Movie 8. There are a few factors contributing to the pulling force required for successful disassembly, namely, the force that overcomes magnetic attraction between the assembly pair, the force to deform the soft segment, and the friction force. To understand the force‒displacement curve during the disassembly process, we show the configurations of the MaSoChain as insets in Fig. 8 at critical time steps and mark the key process of magnetic disassembly and bending of the soft segment with light color blocks that match the force peaks in the disassembly process. Between the peaks, we think the flat curve provides a hint for the local kinetic friction force between the thoracic catheter and the MaSoChain, although it depends on the configurations. We also find that the assembly/disassembly forces depend on the size of the MaSoChain, as shown in Supplementary Figs. 9 and 10.

In the end, we demonstrated a prototype of a self-folding tethered capsule endoscope based on self-folding MaSoChains. One limitation of the existing endoscopes is that all functional components must be integrated at the tip^5,51^ (working channel to pass biopsy, air/water channel, camera, LED, etc.), making it very difficult to miniaturize. However, we think MaSoChain can address this challenge by on-demand folding after passing through the narrow opening in the natural orifice (e.g., nose cannel) and assembling to a fully functional tethered capsule endoscope.

**Fig. 9:**
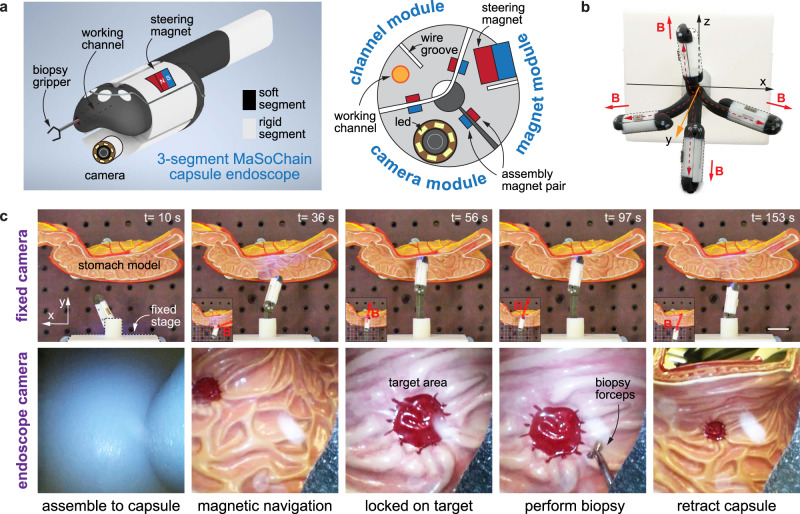
Self-folded tethered capsule endoscope made by three-segment MaSoChain. **a** Structural design of a foldable endoscope with 3 functional segments: the camera module, the magnet module with a large steering magnet and the channel module through which a biopsy gripper can pass. The three-segment MaSoChain structure can self-assemble into a capsule endoscope when pushing out the tube. **b** The tethered capsule endoscope can be actively steered using an external magnetic field. The capsule position and orientation are shown with corresponding magnetic field directions. **c** Demonstration of the use of the MaSoChain endoscope with both the fixed lab camera view (upper row) and the onboard endoscope camera view (lower row). The scale bar is 25 mm. The process included complete assembly of the three-segment capsule endoscope, magnetic navigation of the capsule, locking on the target, insertion of biopsy forceps, performing biopsy, and retraction of the capsule. The complete experiment can be found in Supplementary Movie 9.

We designed this tethered capsule endoscope based on a three-segment MaSoChain. As shown in Fig. 9, each segment contains a functional module in the endoscope: a camera module with integrated LEDs for observation, a magnetic module with a large NdFeB steering magnet for effective magnetic navigation, and a channel module that allows biopsy forceps (Olympus, FB-56D-1, scope channel: 1.2 mm) to pass through to acquire tissue samples. In Fig. 9c, we demonstrate a basic working process of the MaSoChain capsule endoscope using the views of a fixed camera and the onboard endoscope camera. First, the MaSoChain is self-assembled when pushing out of the sheathing tube. Then, an external magnetic field is used to help navigate inside the stomach model and locked on the targeted position. A biopsy forceps is passed through the working channel to perform local tissue sampling, and in the end, the endoscope is retracted. A complete demonstration can be viewed in Supplementary Movie 9.

## Discussion

The results presented here address a fundamental challenge in MIS in that the geometry and functionality are limited by the inner diameter of the catheter sheath. Through a combination of elastic and magnetic energies, we have developed a strategy to synthesize large functional structures at the tip of the catheter by simply pushing and pulling the composite chains. The strategy is not limited by the size, and it is compatible with existing magnetic navigation systems. For the miniaturized design of MaSoChain (<1 mm), one cannot continue to use 3D-printed soft materials (Agilus) due to their insufficient elastic force and torque. The time-dependent viscoelasticity of 3D-printed soft materials is difficult to predict, which makes it challenging to implement for large-scale clinical applications. In the future, one can implement commercially available superelastic nitinol alloy to overcome these limitations and reduce the friction with the sheathing catheter (Supplementary Fig. 11). We believe this strategy can be potentially impactful for a wide range of medical applications with more functional and intelligent tools for future minimally invasive interventions.

## Methods

### 3D printed composite MaSoChains

MaSoChain samples were fabricated using multimaterial 3D printing by the Objet printer (Stratasys Inc.) for both rigid and soft materials. The rigid material (VeroWhite, color: white) has a typical tensile strength of 50–65 MPa, a modulus of elasticity of 2000–3000 MPa, and a 10–25% maximum elongation. The soft material (Agilus30, color: black) is a typical rubber-like hyperelastic material. It has a much lower tensile strength of 2.4–3.1 MPa and a shore hardness (scale A) of only 30 but has a very high elongation at a break of 220–270%, which makes it a good choice for elastic springs.

Small NdFeB magnets (article: 9964-2353, HKCM Engineering) were used to assemble magnets between rigid segments for the samples in Figs. 1–5. These N42 grade magnets have a flux density inside the magnet of 1.28 T, and the dimensions are 1.2 × 1 × 0.5 mm. One magnet can lift a weight of 40 g, which is much higher than the average weight of a chain link of approximately 0.1 g (one hard segment, one soft segment, and two magnets). For the samples in Figs. 6, 7, different cuboid NdFeB magnets were used for assembly. These N52 grade magnets have a flux density inside the magnet of 1.43 T with dimensions of 2 × 2 × 1 mm. One such magnet can lift a weight of 160 g. (article: 9964-66392, HKCM Engineering)

Stronger magnets were used to account for the flexible electronics as well as the increase in size as the cross-section of the rigid segment was increased from 3 × 3 mm (Figs. 1–4) to 5 × 5 mm (Figs. 6, 7). The depth of the groove was dependent on the PCB used. For the samples in Fig. 6, the depth is 3 mm, and for the samples in Fig. 7, the depth is 4 mm. The width of the groove is 0.3 mm for all the samples, which creates a loose fit with the PCB. The three white cylinders have a diameter of 2.3 mm each and extend 1 mm below the PCB. All outer dimensions of the soft segment were upscaled proportionally to the increase in width from 3 mm to 5 mm (from samples in Figs. 1–5 to samples in Figs. 6, 7), except for the inner radius of the soft segment, which was kept constant at 1.15 mm. This results in a cross-section of 4 × 5 mm in the middle of the soft section.

### Characterization of the elasticity of soft segments

We measured the elastic performance of the soft segments (black) through a customized experiment, as shown in Supplementary Fig. 1. The rigid segment connected at one end of the soft segment was glued onto the substrate, while the rest was left free. We fixed three NdFeB magnets (dimension 3.6 × 3.5 × 1.6 mm, Magnet-Cuboid Q03.6 × 03.5 × 01.6Ni-45SH, HKCM Engineering e.K.) inside the rigid segments and applied a slowly varying uniform magnetic field (10 mT, rotating speed 5 degrees per second in the same plane). Because of the slow motion, we ignored the viscoelasticity in the 3D printed material and assumed that the magnetic torque was equal to the elastic torque provided by the soft segment. The magnetic torque can be calculated from the angle difference between the external magnetic field and the orientation of the three NdFeB magnets. The results can be seen in Supplementary Fig. 1c, and the total elastic energy is calculated as in Fig. 2e.

### Magnetic interaction in the assembly process

The magnetic potential energy between each pair of assembly magnets can be modeled by the dipole‒dipole interaction as

1 $$H=-\frac{{\mu }_{0}}{4\pi {\left|{{{{{\bf{r}}}}}}\right|}^{3}}\left[3\left({{{{{{\bf{m}}}}}}}_{{{{{{\bf{1}}}}}}}\cdot \hat{{{{{{\bf{r}}}}}}}\right)\left({{{{{{\bf{m}}}}}}}_{{{{{{\bf{2}}}}}}}\cdot \hat{{{{{{\bf{r}}}}}}}\right)-{{{{{{\bf{m}}}}}}}_{{{{{{\bf{1}}}}}}}\cdot {{{{{{\bf{m}}}}}}}_{{{{{{\bf{2}}}}}}}\right]$$

where ${\mu }_{0}$ is the magnetic constant, $\hat{{{{{{\bf{r}}}}}}}$ is a unit vector parallel to the line connecting the centers of the two dipoles, and $|{{{{{\bf{r}}}}}}|$ is the distance between the centers of ${{{{{{\bf{m}}}}}}}_{{{{{{\boldsymbol{1}}}}}}}$ and ${{{{{{\bf{m}}}}}}}_{{{{{{\bf{2}}}}}}}$. Here, we assume that the magnets are a point dipole, where the dipole moment is at its geometric center. The flux density ${H}_{{cb}}$ is 958 kA m^(−1) (based on the datasheet from HKCM Engineering). Each assembly magnet has a magnetic dipole moment determined by the normal coercivity (${H}_{{cb}}$) and the volume (V) of the magnet: $\left|{{{{{{\bf{m}}}}}}}_{{{{{{\bf{1}}}}}}}\right|$ = $\left|{{{{{{\bf{m}}}}}}}_{{{{{{\bf{2}}}}}}}\right|$ = m*V*. If we assume that the potential energy of two infinitely far magnetic dipoles is zero, the minimum magnetic energy is at $\left|{{{{{\bf{r}}}}}}\right|$ = 0.0005 m, which is equal to H_min = −5^(−4) J (rough number from Fig. 2).

The relative position of both assembly magnet pairs was determined through an experiment. As shown in Supplementary Fig. 1a, b, the central position and orientation of the rigid object (Supplementary Fig. 1d) were tracked and plotted. As a result, it was possible to reconstruct the trajectory of the assembly magnets during the folding process based on the relative position r between two pairs of assembly magnets. We numerically calculated the magnetic energy under different theta angles in Fig. 2d, e.

### Design of flexible electronics

A total of six different PCBs were designed for this project with similar flexible PCB properties. The flexible PCBs consist of a 25 µm polyimide core, two 18 µm copper layers (0.5 oz), and a 27.5 µm yellow overlay (Polyimide incl. adhesive), which sum up to a total PCB thickness of 116 µm. Exposed pads have a surface finish of 1U” immersion gold (ENIG) to improve solderability and connectivity of connecting pads, as highlighted in Fig. 6d. In general, traces have a minimum width of 0.254 µm (10 mils). The width is decreased for heating patterns that have a trace width of 76 µm (3 mils). The trace minimum spacing for all PCBs is 76 µm (3 mils). To prevent a potential fracture of the traces while bending, all traces were perpendicularly oriented to the bending edges.

The PCBs in Fig. 6 have 4 LEDs that were connected in parallel, and each leading resistance was chosen such that the circuit could be connected to a 5 V power source. The heating pattern dimensions were designed for a maximum current of 300 mA, at which the temperature rises to 60 °C, which is also the maximum operating temperature for the magnets used. The total resistance mainly depends on the length of the 3 mm traces. Therefore, the letter “E” from Fig. 7 has the highest total resistance of 14.5 Ohm, followed by the letter “T” with 11 Ohm and the letter “H” with 10.5 Ohm. All resistances were measured at room temperature.

### Modeling of the magnetic force between two NdFeB assembly magnets

The two assembly magnets are in close contact with each other during the assembly and disassembly process. The geometry of the assembly magnets needs to be considered for force estimation. This means that we cannot use a simplified point-based dipole‒dipole interaction model for the magnetic interactions because they rely on the assumption that the distance between two dipoles is much larger than the magnet size.

To include the effect of the geometries, we wrote a MATLAB script to calculate the interactions between the two assembly magnets (block NdFeB: 1.2 × 1 × 0.5 mm, dipole direction is along the 0.5 mm axis) based on the finite element method, where we consider each assembly as a small number ($o\times p\times q$) of unit magnets at different locations. Then, we can represent the interaction between the two large assembly magnets as a summation of dipole‒dipole interactions between each small magnet pair at its own geometric center (Supplementary Fig. 4a). This method can effectively include the geometric factor of the assembly magnets, providing an accurate estimation of force in the disassembly process.

Magnetic dipole‒dipole force:

2 $${{{{{{\bf{F}}}}}}}_{{m}_{1}-{m}_{2}}=	\frac{3{\mu }_{0}}{4\pi {\left|{{{{{\bf{r}}}}}}\right|}^{4}}\left\{\left(\hat{{{{{{\bf{r}}}}}}}\times {{{{{{\bf{m}}}}}}}_{1}\right)\times {{{{{{\bf{m}}}}}}}_{2} \right.\\ 	 \left.+\left(\hat{{{{{{\bf{r}}}}}}}\times {{{{{{\bf{m}}}}}}}_{2}\right)\times {{{{{{\bf{m}}}}}}}_{1}-2\hat{{{{{{\bf{r}}}}}}}\left({{{{{{\bf{m}}}}}}}_{1}\cdot {{{{{{\bf{m}}}}}}}_{2}\right)+5\hat{{{{{{\bf{r}}}}}}}[(\hat{{{{{{\bf{r}}}}}}}\times {{{{{{\bf{m}}}}}}}_{1})\cdot (\hat{{{{{{\bf{r}}}}}}}\times {{{{{{\bf{m}}}}}}}_{2})]\right\}$$

Magnetic energy potential between two point dipoles:

3 $${H}_{{m}_{1}-{m}_{2}}=-\frac{{\mu }_{0}}{4\pi {\left|{{{{{\bf{r}}}}}}\right|}^{3}}\left[3\left({{{{{{\bf{m}}}}}}}_{1}\cdot \hat{{{{{{\bf{r}}}}}}}\right)\left({{{{{{\bf{m}}}}}}}_{2}\cdot \hat{{{{{{\bf{r}}}}}}}\right)-{{{{{{\bf{m}}}}}}}_{1}\cdot {{{{{{\bf{m}}}}}}}_{2}\right]-{\mu }_{0}\frac{2}{3}{{{{{{\bf{m}}}}}}}_{1}\cdot {{{{{{\bf{m}}}}}}}_{2}\delta ({{{{{\bf{r}}}}}})$$

Total force between two assembly magnets:

4 $${{{{{{\bf{F}}}}}}}_{{total}}=\mathop{\sum }\limits_{i=1}^{i}\mathop{\sum }\limits_{j=1}^{j}{{{{{{\bf{F}}}}}}}_{{m}_{i}-{m}_{j}}$$

Total magnetic potential energy between two assembly magnets:

5 $${H}_{{total}}=\mathop{\sum }\limits_{i=1}^{i}\mathop{\sum }\limits_{j=1}^{j}{H}_{{m}_{i}-{m}_{j}}$$

Based on the equation and method mentioned above, we calculated the magnetic force between two assembly magnets (numbers 1 and 2) with different relative positions. As shown in Supplementary Fig. 4b, we fixed the central position of magnet number 2 at position P_2_(−0.5,0,0) (unit: mm). We move the central position of magnet number 1 in the workspace and calculate the corresponding magnetic force and energy. P_1_(x,y,z) represents the central position of magnet number 1. The scanning volume is marked in Supplementary Fig. 4b, from 0 to 3 mm in the x-direction, −3 to 3 mm in the y-direction, and −3 to 3 mm in the z-direction. We also use the same coordinates for plotting the calculated results.

### Stability analysis of the assembly magnet pair near a large magnet

We provide an analytical model to show that disassembling an assembly pair using a large external magnet is very unlikely. We first consider a simple case, and then we generalize the case for more complex configurations.

In the simple case, we fix one NdFeB assembly magnet (1.2 × 1 × 0.5 mm) where its center position is at the origin of the Cartesian coordinate system (x = y = z = 0). Its dipole position points toward the positive x direction (${{{{{{\bf{x}}}}}}}_{1}={[{{{{\mathrm{0,0,0}}}}}]}^{T}$ with magnetic dipole moment ${{{{{{\bf{m}}}}}}}_{1}={[{m}_{1},{{{{\mathrm{0,0}}}}}]}^{T}$). Now, we consider placing a dipole magnet on the left in the x coordinate (position: ${{{{{{\bf{x}}}}}}}_{2}={[-L,{{{{\mathrm{0,0}}}}}]}^{T}$, with magnetic dipole moment ${{{{{{\bf{m}}}}}}}_{2}={[{m}_{2},{{{{\mathrm{0,0}}}}}]}^{T}$), as shown in Supplementary Fig. 7a.

The magnetic force between the two dipole magnets is:

6 $${{{{{{\bf{F}}}}}}}_{{m}_{1}-{m}_{2}}=	\frac{3{\mu }_{0}}{4\pi {\left|{{{{{\bf{r}}}}}}\right|}^{4}}\left\{\left(\hat{{{{{{\bf{r}}}}}}}\times {{{{{{\bf{m}}}}}}}_{1}\right)\times {{{{{{\bf{m}}}}}}}_{2}+\left(\hat{{{{{{\bf{r}}}}}}}\times {{{{{{\bf{m}}}}}}}_{2}\right)\times {{{{{{\bf{m}}}}}}}_{1}-2\hat{{{{{{\bf{r}}}}}}}\left({{{{{{\bf{m}}}}}}}_{1}\cdot{{{{{{\bf{m}}}}}}}_{2}\right) \right.\\ 	 \left.+5\hat{{{{{{\bf{r}}}}}}}[(\hat{{{{{{\bf{r}}}}}}}\times {{{{{{\bf{m}}}}}}}_{1})\cdot (\hat{{{{{{\bf{r}}}}}}}\times {{{{{{\bf{m}}}}}}}_{2})]\right\}$$

where ${\mu }_{0}$ is the magnetic permeability in a vacuum and ${{{{{\bf{r}}}}}}$ is the relative position vector between the centers of the two dipole moments ${{{{{{\bf{m}}}}}}}_{1}$ and ${{{{{{\bf{m}}}}}}}_{2}$.

7 $${{{{{\bf{r}}}}}}={{{{{{\bf{x}}}}}}}_{1}-{{{{{{\bf{x}}}}}}}_{2}={[L,0,0]}^{{{{{{\rm{T}}}}}}}$$

After plugging in all the relevant values, the force between two magnets can be expressed as

8 $${{{{{{\bf{F}}}}}}}_{{m}_{1}-{m}_{2}}=\frac{3{\mu }_{0}}{4\pi {\left|{{{{{\bf{r}}}}}}\right|}^{4}}{m}_{1}{m}_{2}\left[\begin{array}{c}1\\ 0\\ 0\end{array}\right]$$

Now let us consider that there are two magnets with opposite dipole directions, as shown in Supplementary Fig. 7b. In this case, two magnets (${{{{{{\bf{m}}}}}}}_{2}$ and ${{{{{{\bf{m}}}}}}}_{3}$) generate opposite force directions on magnet ${{{{{{\bf{m}}}}}}}_{1}$. We would like to use this case to find the scaling effect of magnet ${{{{{{\bf{m}}}}}}}_{3}$ that can destabilize the assembly pair ${{{{{{\bf{m}}}}}}}_{1}$ and ${{{{{{\bf{m}}}}}}}_{2}$.

If we consider both ${m}_{1}$ and ${m}_{2}$ to be NdFeB assembly magnets (1.2 × 1 × 0.5 mm), then we consider the third magnet with an opposite dipole direction with magnetic moment ${{{{{{\bf{m}}}}}}}_{3}={[{-m}_{3},{{{{\mathrm{0,0}}}}}]}^{T}$, which provides a repulsion force. If we consider that ${{{{{{\rm{m}}}}}}}_{3}$ is sufficiently large that the force can balance the attraction force for ${{{{{{\rm{m}}}}}}}_{2}$, it needs to be balanced; as a result:

9 $${{{{{{\bf{F}}}}}}}_{{m}_{1}-{m}_{2}}+{{{{{{\bf{F}}}}}}}_{{m}_{1}-{m}_{3}}=0$$

10 $$\frac{3{\mu }_{0}}{4\pi {L}^{4}}{m}_{1}{m}_{2}-\frac{3{\mu }_{0}}{4\pi {D}^{4}}{m}_{1}{m}_{3}=0$$

The required ${{{{{{\rm{m}}}}}}}_{3}$ needs to be large as ${m}_{3}=\frac{{D}^{4}}{{L}^{4}}{m}_{2}$at position ${{{{{{\bf{x}}}}}}}_{3}={[-D,{{{{\mathrm{0,0}}}}}]}^{T}$ to balance the magnetic force generated by ${{{{{{\rm{m}}}}}}}_{2}$. Now, if we assume that ${{{{{{\rm{m}}}}}}}_{3}$ is a cube magnet with an edge size of a, we can rewrite the equation as

11 $${M}_{{NdFeB}}{a}^{3}=\frac{{D}^{4}}{{L}^{4}}{m}_{2}$$

where ${M}_{{NdFeB}}$ is the magnetization of the NdFeB magnet, which is equal to $1.08\times {10}^{6}$ A m^(-1). If we now consider D as a variable and consider how a needs to scale with D, we will find

12 $$a=\root 3 \of {{\frac{{m}_{2}}{{M}_{{NdFeB}}{L}^{4}}}}{D}^{\frac{4}{3}}$$

It shows that with an increasing distance D, the third magnet needs to increase rapidly in size (${ \sim D}^{\frac{4}{3}}$) to match the force. This means that the magnet needs to be larger than a to destabilize the assembly pair between ${{{{{{\rm{m}}}}}}}_{1}$ and ${{{{{{\rm{m}}}}}}}_{2}$. If the ${{{{{{\rm{m}}}}}}}_{2}$ magnet is touching ${{{{{{\rm{m}}}}}}}_{1}$, as in the assembly pair, the required size of ${{{{{{\rm{m}}}}}}}_{3}$ can be so large that it is physically impossible to fit on the left side. If one changes the ${{{{{{\rm{m}}}}}}}_{3}$ direction, it will only decrease the magnetic repulsion force. The above scaling law provides an important insight that the magnetic gradient generated by a nearby permanent magnet is very unlikely to destabilize the magnetic assembly pair. The analysis results also resonate with our experimental observations. Therefore, we can conclude that the assembly will be stable in our envisioned applications regardless of the neighboring NdFeB magnet configurations.

### Force measurement

To measure the force required for pushing and pulling of the MaSoChain, three-segmented MaSoChains were prepared in four different sizes. (i.e., the cross-sectional square side length: 3.4, 3.6, 3.8, and 4.0 mm.). A commercial thoracic catheter was purchased from Primed (Germany), which is a flexible tube with a 6 mm inner diameter. A moistened MaSoChain was placed inside the catheter to mimic a similar situation in use. Then, the MaSoChain was joined to the force sensor (nano 17, ATI Industrial Automation, USA) assembled to a linear motorized stage (Supplementary Fig. 8). The stage moved at a speed of 5 mm s^(−1), and DAQ software recorded force data at a speed of 1 kHz. The force signals and videos were synced for analysis.

### Image and video acquisition

All images and videos were acquired using Fujifilm X-T20, Fujifilm X-T4, and Sony DSC-RX100 III under soft lighting conditions. Unless specified, all videos are played in real-time. Parts of Fig. 1b were drawn by using pictures from Servier Medical Art. Servier Medical Art by Servier is licensed under a Creative Commons Attribution 3.0.

## Supplementary information

Supplementary Information

Peer Review File

Description of Additional Supplementary Files

Supplementary Movie 1

Supplementary Movie 2

Supplementary Movie 3

Supplementary Movie 4

Supplementary Movie 5

Supplementary Movie 6

Supplementary Movie 7

Supplementary Movie 8

Supplementary Movie 9

## Data Availability

All data are available in the main text or supplementary materials.

## Code availability

All the relevant code used to generate the results in this paper and the Supplementary Information is available upon request.
