# Supplementary material for: Self-folding soft-robotic chains with reconfigurable shapes and functionalities
Source: Nat Commun. 2023 Mar 7;14:1263. doi: 10.1038/s41467-023-36819-z (PMC9992713; doi:10.1038/s41467-023-36819-z)
Supplement: Supplementary file 1 — Supplementary Information [file 41467_2023_36819_MOESM1_ESM.pdf]

Supplementary Information

Self-folding soft-robotic chains with reconfigurable shapes and functionalities

Hongri Gu[1,2]*, Marino Möckli[1], Claas Ehmke[1], Minsoo Kim[1]*, Matthias Wieland[1], Simon Moser[1], Clemens Bechinger[2], Quentin Boehler[1]*, Bradley J. Nelson[1]*

1, Institute of Robotics and Intelligent Systems, ETH Zurich, Zurich, Switzerland

2, Department of Physics, University of Konstanz, Konstanz, Germany

*Correspondence to: hongri.gu@uni-konstanz.de, minkim@ethz.ch, qboehler@ethz.ch, and bnelson@ethz.ch

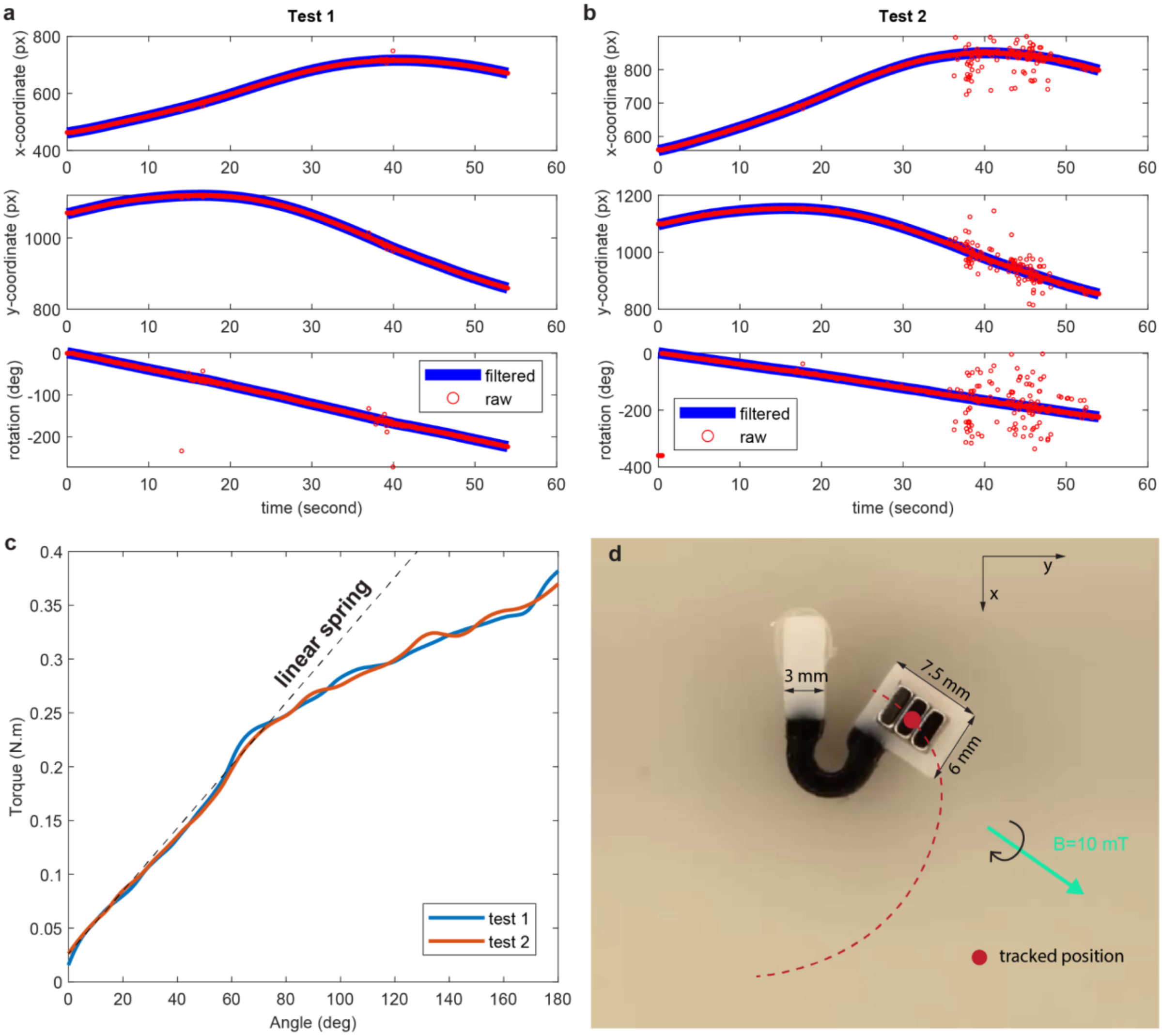

Supplementary Fig. 1 | Position and orientation tracking results of the characterization of the elasticity of the soft robots. a, b Tracked position and orientation of the free end of the soft segment. **c** Diagram of elastic torque versus bending angle. **d** A snapshot of the bending experiments for the soft elastic segment.

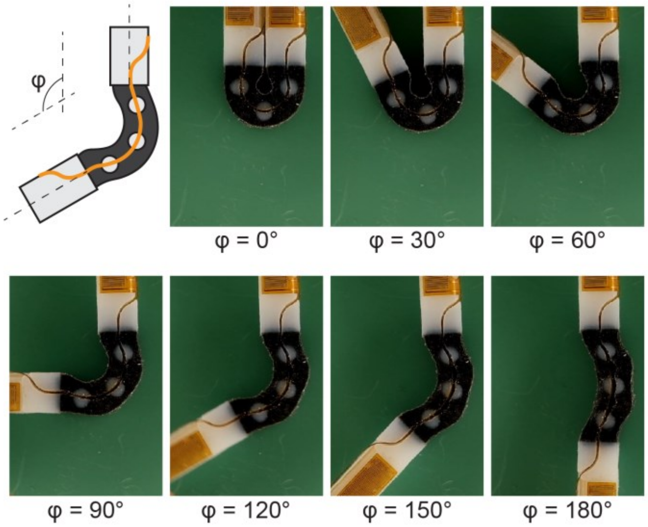

Supplementary Fig. 2 | Soft elastic segment with round pins at different angles. The embedded rigid pins (three white cylinders) hold the soft elastic segment together with a slit in the middle to pass the flexible PCB at different angles (φ from 0 to 180 degrees). Without the pins, the soft segment would separate into two parts under bending, which would significantly change its elasticity.

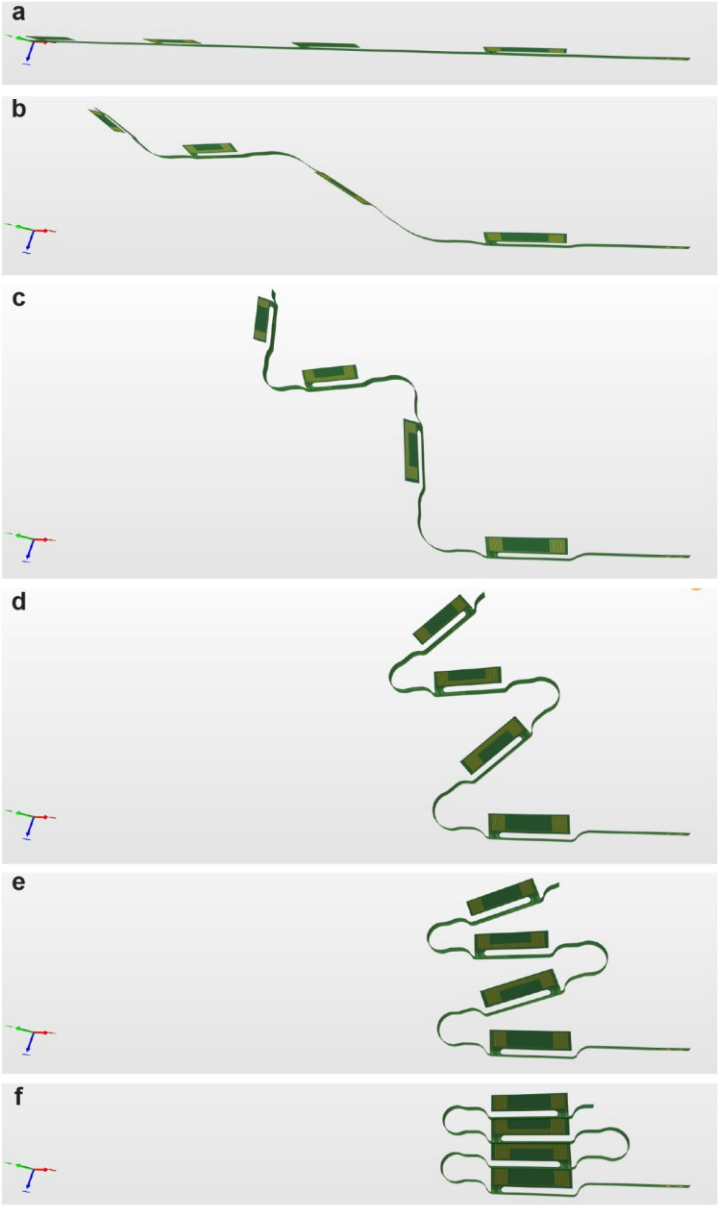

Supplementary Fig. 3 | The complete folding process of the flexible printed circuit board (PCB) for the programmable heating surface.

a

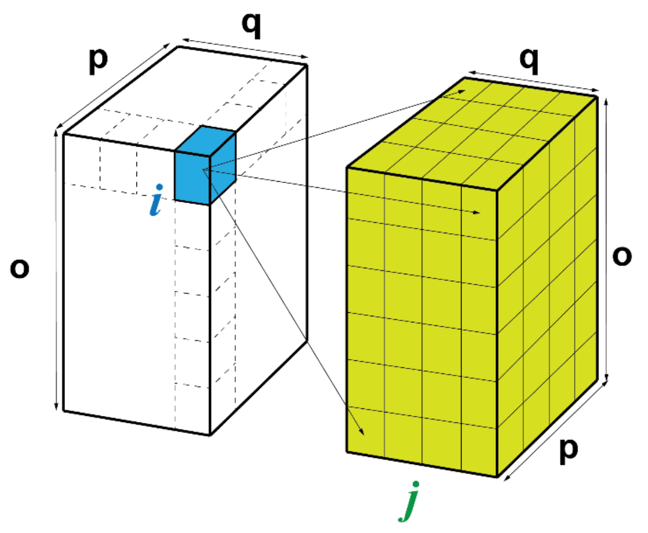

b

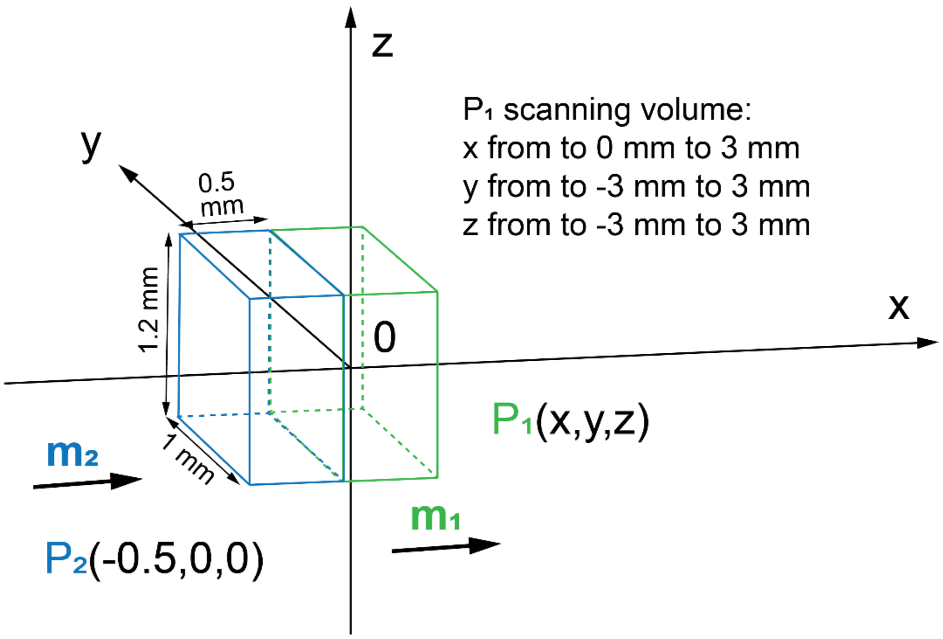

Supplementary Fig. 4 | a Illustration of a finite element method to calculate the magnetic force and potential between two close block magnets**. b** Illustration of the coordinate system and the relative positions between two assembly magnets. Magnet number 2 is fixed with central position P2(-0.5,0,0), and P1(x,y,z) scanning in the workspace.

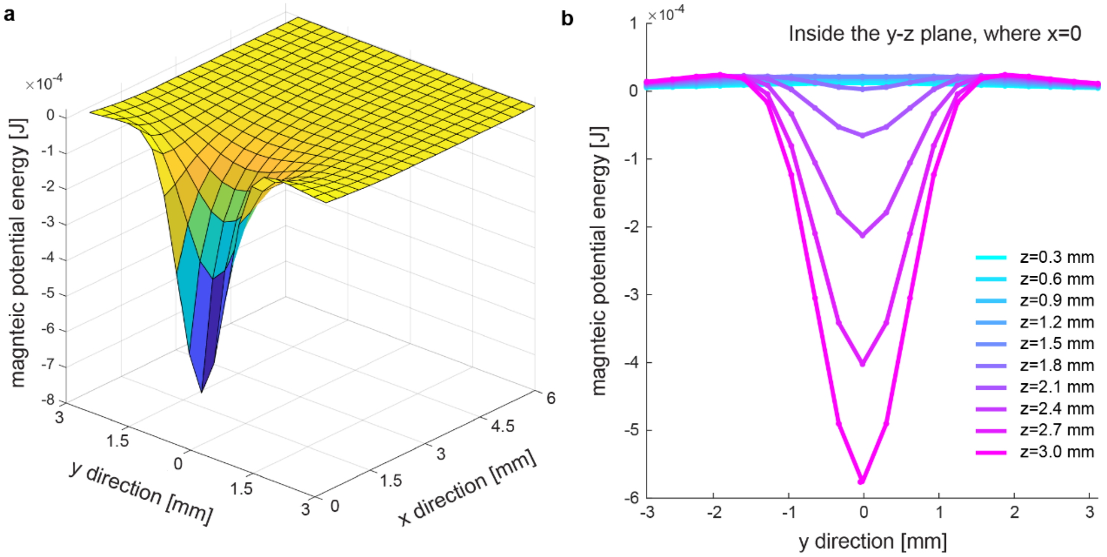

Supplementary Fig. 5 | Calculated magnetic potential between the two NdFeB assembly magnets. a Magnetic energy between the two magnets inside z=0 plane, with P1 scan 0 to 3 mm in x direction and -3 to 3 mm in the y direction. **b** The magnetic energy between two magnets inside the y-z plane where x=0 mm. The coordinates and configurations can be found in Supplementary Fig. 4b.

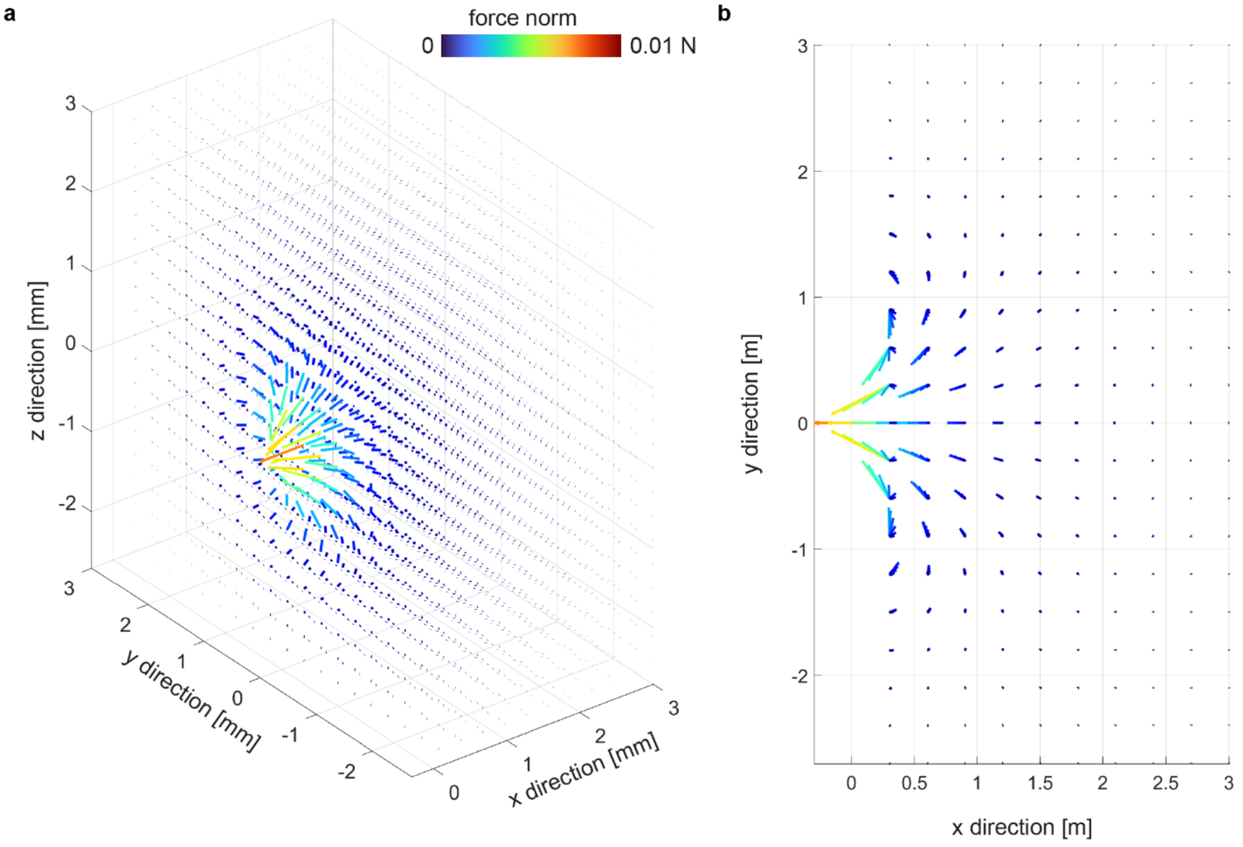

Supplementary Fig. 6 | Simulated assembly force between the assembly pair magnets (two NdFeB magnets with the same dipole direction). Bird view and top view of the forces in the workspace are shown in panel a and b, respectively. The original configuration of the simulated configurations can be found in Supplementary Fig. 4b.

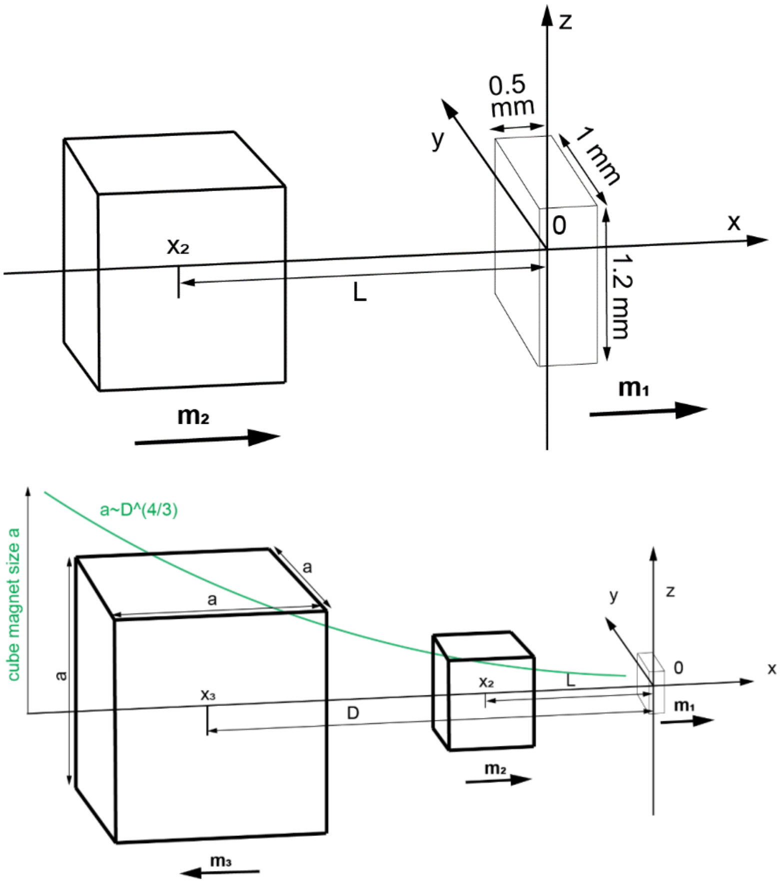

Supplementary Fig. 7 | Force analysis of two competing magnets with opposite dipole directions at different distances.

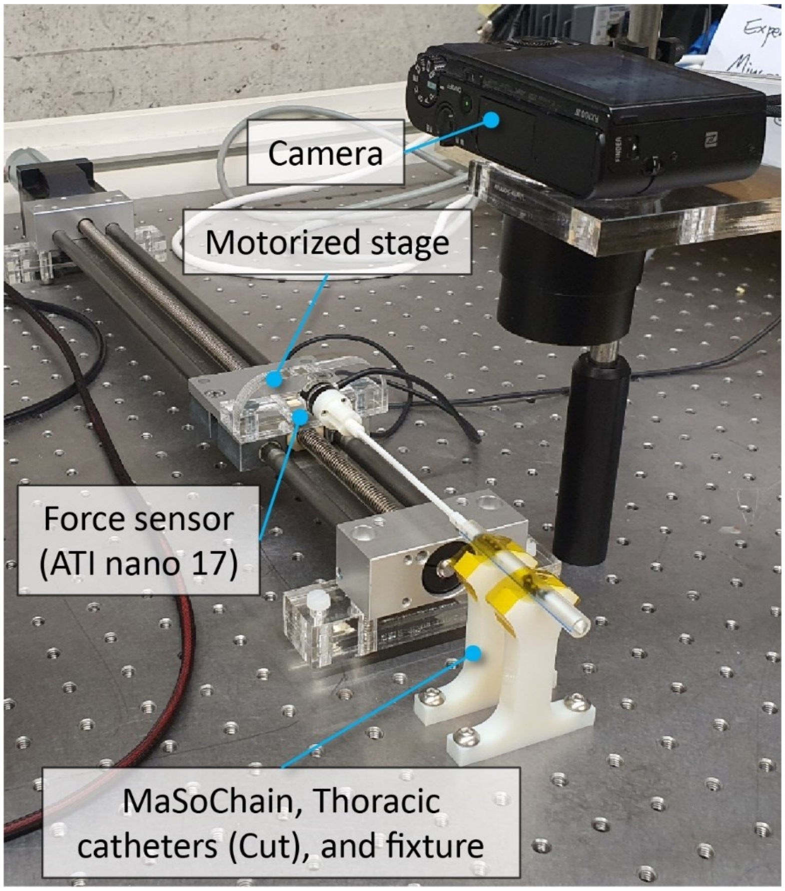

Supplementary Fig. 8 | Experimental force measurement setup. The motion of the MaSoChain is controlled by a motorized stage at a constant speed. The force along the axial direction between the MaSoChain and the thoracic catheters is measured.

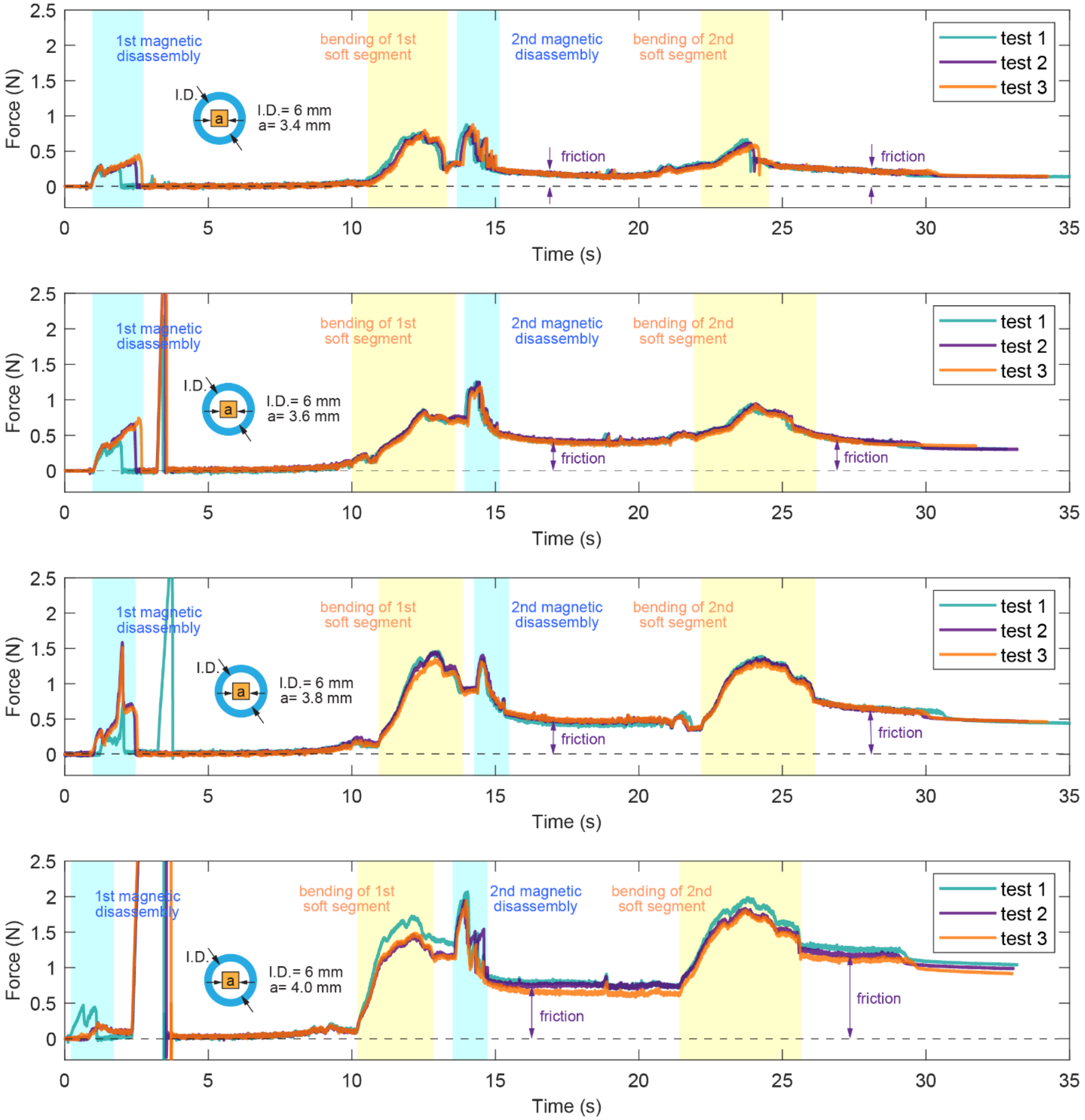

Supplementary Fig. 9 | The complete force measurement results between a three-segment MaSoChain and medical grade thoracic catheter during the disassembly process. Four MaSoChain samples are tested with different cross-section dimensions (square length: 3.4 mm, 3.6 mm, 3.8 mm, and 4.0 mm). The 24 French thoracic catheter has an inner diameter of 6 mm. The pulling speed is 5 mm per second. The pulling force along the axial direction is measured with a 1k sampling rate.

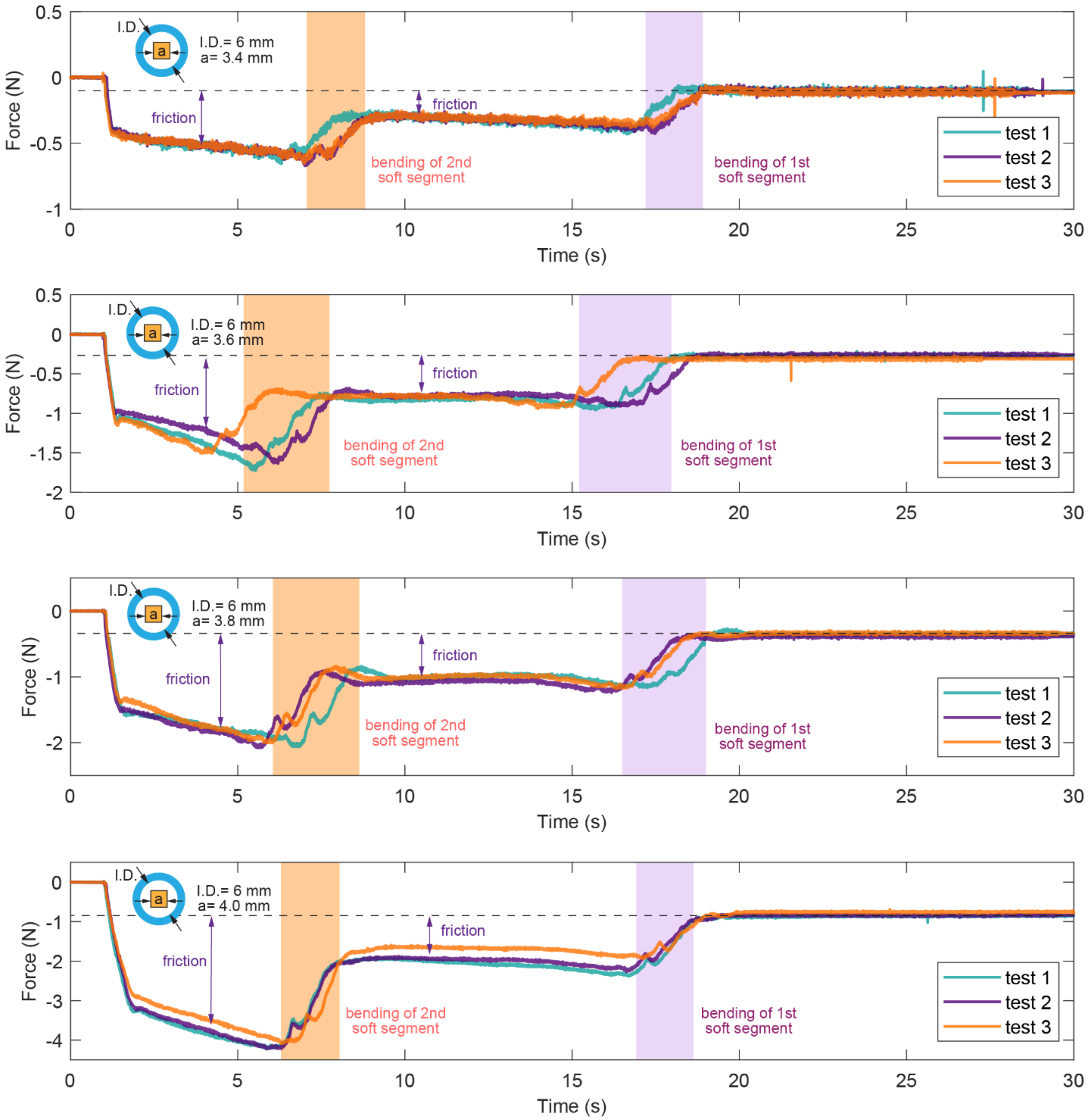

Supplementary Fig. 10 | The complete force measurement results between a three-segment MaSoChain and medical grade thoracic catheter during the assembly process. Four MaSoChain samples are tested with different cross-section dimensions (square length: 3.4 mm, 3.6 mm, 3.8 mm, and 4.0 mm). The 24 French thoracic catheter has an inner diameter of 6 mm. The pushing speed is 5 mm per second. The pushing force along the axial direction is measured with a 1k sampling rate.

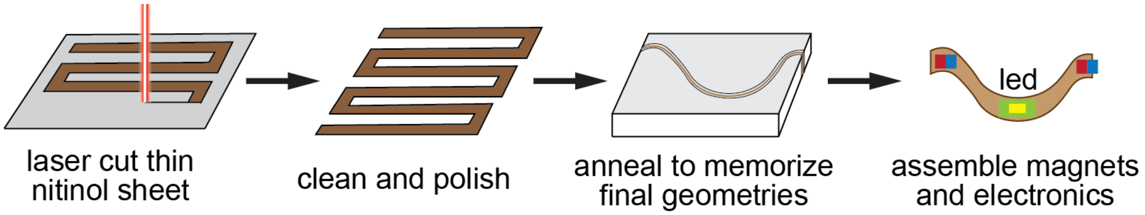

Supplementary Fig. 11 | A potential method to miniaturize MaSoChain to the submillimeter scale using nitinol. In this proposed fabrication process, we first design and use a femtosecond laser to cut customized stripes from a nitinol thin film. The film is then cleaned, polished and put into a customized mold for the final folded geometry, and the metal mold and nitinol stripe are then placed in the oven for annealing. After cooling to room temperature, electronics and magnets are assembled onto the MaSoChain, and some coating may be needed to protect the electronics. We think this method might be useful for fabricating MaSoChains with a diameter smaller than 0.5 mm.
