# Supplementary material for: Self-folding soft-robotic chains with reconfigurable shapes and functionalities
Source: Nat Commun. 2023 Mar 7;14:1263. doi: 10.1038/s41467-023-36819-z (PMC9992713; doi:10.1038/s41467-023-36819-z)
Supplement: Supplementary file 3 — Description of Additional Supplementary Files [file 41467_2023_36819_MOESM3_ESM.pdf]

Description of Additional Supplementary Files

Supplementary Movie 1: Self-folding MaSoChains assemble into complex 2D geometries

Supplementary Movie 2: Self-folding MaSoChains assemble into 3D geometries

Supplementary Movie 3: Expanded accessible regions for the tip through a 2-stage self-folding

Supplementary Movie 4: Demonstration of a large gripper folded by a MaSoChain

Supplementary Movie 5: A self-folding MaSoChain with integrated LEDs on a single strip of flex PCB

Supplementary Movie 6: Shape-sensing MaSoChain indicated by LEDs

Supplementary Movie 7: Demonstration of a large heating surface with programmable heating patterns ETH

Supplementary Movie 8: Assembly and disassembly force characterizations inside a thoracic catheter

Supplementary Movie 9: A self-folded tethered capsule endoscope based on three-segment MaSoChains
