# Supplementary material for: Self-folding soft-robotic chains with reconfigurable shapes and functionalities
Source: Nat Commun. 2023 Mar 7;14:1263. doi: 10.1038/s41467-023-36819-z (PMC9992713; doi:10.1038/s41467-023-36819-z)
Supplement: Supplementary file 2 — Peer Review File [file 41467_2023_36819_MOESM2_ESM.pdf]

nature portfolio

Peer Review File

Self-folding soft-robotic chains with reconfigurable shapes and functionalities

Open Access This file is licensed under a Creative Commons Attribution 4.0 International License, which permits use, sharing, adaptation, distribution and reproduction in any medium or format, as long as you give appropriate credit to the original author(s) and the source, provide a link to the Creative Commons license, and indicate if changes were made. In the cases where the authors are anonymous, such as is the case for the reports of anonymous peer reviewers, author attribution should be to 'Anonymous Referee' followed by a clear attribution to the source work. The images or other third party material in this file are included in the article's Creative Commons license, unless indicated otherwise in a credit line to the material. If material is not included in the article's Creative Commons license and your intended use is not permitted by statutory regulation or exceeds the permitted use, you will need to obtain permission directly from the copyright holder. To view a copy of this license, visit http://creativecommons.org/licenses/by/4.0/.

REVIEWER COMMENTS

Reviewer #1 (Remarks to the Author):

This work presents an interesting method that may partly overcome the limitations of existing surgical soft robots. The reconfigurable structure possesses a possibility that surgical tools can be delivered through a narrow catheter by unfolding the structure into line segments. Despite the promise of the work, there are a number of major questions and concerns that the authors should address before I could make a recommendation on this paper.

0. This paper starts with a comparison between the proposed mechanism and the other reconfigurable mechanisms. The author claims that current issues in the reconfigurable soft robots can be resolved by adopting the proposed mechanism. However, this paper does not clearly demonstrate that MaSoChain can overcome the limitations. The proposed devices are just conceptual applications of the mechanism that cannot perform tasks that are required in a real situation. For example, the ‘accessible region 2’ in Fig. 4 does not have any meaning in terms of the workspace of the device. In a more realistic situation, the accessible region 2 can be easily reached by just retracting the catheter a little bit. The accessible region 2 is then perfectly covered by the accessible region 1. In addition, it is unclear what the author would like to demonstrate with the gripper in Fig. 5. Is transportation of the cargo actually needed in a real situation? In my opinion, more realistic applications should be proposed to prove the advantage of this mechanism over other reconfigurable mechanisms.

1. The whole claim of this paper is based on the assumption that the MaSoChain is compatible with a catheter, not a plastic tube. A catheter first delivers the MaSoChain to the target position, and the MasoChain is then reconfigured into a larger shape. If this MaSoChain is not compatible with the commercial catheters, the MaSoChain cannot even be delivered to the target position, and the practicality of this concept is not guaranteed. However, it appears to me that the author did not conduct the experiment with a commercial catheter. In particular, the supporting sheath in Fig. 5 appears to be a very stiff catheter or a plastic tube, and the MaSoChain is moved from one position to another by literally moving the whole tube with a human hand which is infeasible in realistic cases. Is this MaSoChain compatible with a commercial catheter?

2. The pulling process of this MaSoChain will apply some amount of force on the tip of the catheter. The force between the two coupled magnets might be enough to flatten the tip of the catheter which is undesirable consequences. The deformed catheter can block the deployment of other devices and the sharpened tip might leave scratches on the surface of organs. Does the force between the magnets cause any deformation on the tip of the commercial-grade catheters?

3. Once the soft robot is reconfigured into a larger shape, the soft segment located in the vicinity of the catheter tip must withstand the weight of the deployed tool. Otherwise, the deployed device will be bent toward the direction of gravity which makes it difficult to control. Does this concentrated weight of the folded structure cause the sag of the tool when it is deployed in 3d space? In addition, can these tools be further advanced from the catheter tip without the sag? This paper only shows cases where the deployed tools are actuated near the tip of a catheter.

Reviewer #2 (Remarks to the Author):

The paper presents a novel solution for assembling large 2D and 3D structures inside the body cavities thanks to self-folding magnets. The idea behind the paper is clever and the fabrication modalities are accessible. In addition, the videos are very interesting and well explain the obtained results.
The reviewer is very positive towards the paper.
On the other hand, the reviewer thinks that the paper could be improved with i) some more details about the pulling forces needed to disassembly to structure; ii) a retuning of some references of the state of the art; iii) a clearer explanation of the effect of external magnetic fields and of the extra magnets.
More details are reported below:
i) The delivery of the structures from the catheter should be straightforward, even if the speed of delivery is not analysed. The reviewer thinks that the assembly could be less or more effective depending on the speed of delivery. Anyhow the most relevant issue is the retrieve of the structure: which are the forces necessary for pulling back the structure and disassemble it? Which is the risk of getting stuck at a certain point at the tip? Can an external magnetic field help to disassembly the structure in case of problems (e.g. by producing a sort of opposite field on to the small magnets)?
In addition, friction during the structure delivery and pulling could be relevant, but the friction problem is never mentioned, except in the last lines ("In the future, one can implement commercially available superelastic nitinol alloy to overcome these limitations and reduce the friction with the sheathing catheter").
ii) When the authors mention the state of the art of reconfigurable robots for surgery and intervention, some seminal works on magnetic retractors, magnetic frames, magnetic cameras could be mentioned. In those cases the access for the magnets is from the mouth or from the abdominal trocar. So the size is larger, but the concept is not far from what the authors are presenting here (e.g. see G. Tortora, 2014, IEEE ASME TMECH).
iii) While the part related to the pure assembly by magnetic forces and elastic energy is clear, when the authors describe the bending and locomotion based on magnetic fields applied externally, the paper leaves some open issues and generates doubts. Is there any risk that the extra bar magnet introduces instabilities in the assembly phase? Are some constraints to be respected in the positioning of the extra magnets to limit instability and/or the generation of secondary structures?

Reviewer #3 (Remarks to the Author):

The manuscript by Hongri Gu et al. describes semisoft inkjet 3D printed structures consisting of elastomeric hinges and rigid material parts with an average cross section of strands approximately 10 x 10 mm2. These structures were equipped with locking NdFeB magnets within the rigid parts and flexible printed circuit boards carrying LEDs and heater. The fabricated 3D structures were unwrapped into a chain and inserted into a tubular sheath for further deployment. The structures were deployed by pushing them gradually out of the sheath. Such structures regain the original 3D shape during the deployment process due to the stress stored in the elastomeric parts, while the integrated magnets lock the rigid parts in their correct place preserving the original 3D shape. The integrated flexible electronic circuits

bring additional benefits to the structure.
The work has a certain level of novelty and can be potentially interesting for a broad readership. The paper can be accepted after the following points have been addressed:

1) To my knowledge atleast one similar approach of magnetic 3D assembly from single strands have been already envisioned previously in e.g. https://doi.org/10.1073/pnas.1910332116 and need to be properly cited.

2) The Authors discuss miniaturization of the fabricated devices several times within the manuscript, often telling that the strategy in not limited by size and comparing their structures with superplastic preformed nitinol medical tools. It would be beneficial for the broad readership to understand how actually one can achieve miniature (cross-sections < 1 mm) self-folding chains offering shape recovery, magnetic locking and integrated electronic circuits? What kind of methods, materials and technologies could be potentially explored to achieve this? The reader expects to see at least a sketch and some discussions on this matter.

3) Shape sensing with LEDs is not completely clear. First of all, a better schematic and the circuit diagram is required explaining this dynamic process. To my opinion the LEDs can indicate only a few locked states but cannot provide a continuous shape sensing function in the broad meaning, which would be of a great importance for the envisioned application scenario. Please clarify how this approach should assist a surgeon in shape sensing when there is no optical observation feasible, for instance during invasive interventions. Please expand your discussion providing alternative solutions.

4) Although it is claimed that the orientation of the locking magnets has been chosen in such a way to cancel the overall torques in external homogeneous magnetic field, it is obvious that no perfect parallel alignment of the magnets can be achieved due to e.g. the nonideal rigidity of the magnet holding structures, 3D printing tolerances and actual magnetization process of the magnets. Du to these sources of misalignment it is impossible to achieve a full compensation of the overall moment for the pair of magnets. Could you please provide estimates and analysis on how these tolerances affect the shape recovery process, locking and actuation in external homogeneous field for at least three orthogonal field orientations with respect to the structure? Please expand the discussion on this matter.
5) There is a certain level of concern about the biocompatibility of the applied materials. Please expand the text addressing the biocompatibility aspects.

6) Please add scalebars in Fig. 1, 4, 6, 7.

RESPONSES TO REVIEWERS' COMMENTS

Reviewer #1 (Remarks to the Author):

This work presents an interesting method that may partly overcome the limitations of existing surgical soft robots. The reconfigurable structure possesses a possibility that surgical tools can be delivered through a narrow catheter by unfolding the structure into line segments. Despite the promise of the work, there are a number of major questions and concerns that the authors should address before I could make a recommendation on this paper.

We appreciate the positive evaluations of our work, with many critical questions, constructive comments, and inspiring suggestions. We have carefully considered those comments and performed additional studies with both experiments and simulations. We hope the substantial data and our answers can address your concerns. Please find the detailed responses as follows:

0. This paper starts with a comparison between the proposed mechanism and the other reconfigurable mechanisms. The author claims that current issues in the reconfigurable soft robots can be resolved by adopting the proposed mechanism. However, this paper does not clearly demonstrate that MaSoChain can overcome its limitations.

We would like to thank this reviewer for this insightful comment, giving us the opportunity to clarify some essential aspects and prevent potential misunderstandings. In this work, we implement a method to fold soft chain structures for large and stable assemblies. This method has a few distinct advantages compared to the previously proposed reconfigurable soft robots at the same scales: (1) large structures with programmable geometries, (2) stable configurations due to the small locking NdFeB magnets, and (3) a simple shape changing mechanism (pull and push the MaSoChain in a tube) without integrating multiple actuators.

These advantages enable the MaSoChains to overcome the limitations of previously proposed shape-reconfiguration methods, and we have demonstrated clearly in Fig. 2 (stable configuration, assembly torque at different folding stages) and Fig. 3 (programmable geometries in 2d and 3d).

The proposed devices are just conceptual applications of the mechanism that cannot perform tasks that are required in a real situation. For example, the 'accessible region 2' in Fig. 4 does not have any meaning in terms of the workspace of the device. In a more realistic situation, the accessible region 2 can be easily reached by just retracting the catheter a little bit. The accessible region 2 is then perfectly covered by the accessible region 1. In addition, it is unclear what the author would like to demonstrate with the gripper in Fig. 5. Is transportation of the cargo actually needed in a real situation? In my opinion, more realistic applications should be proposed to prove the advantage of this mechanism over other reconfigurable mechanisms.

We think that the MaSoChain prototypes presented in this paper can be further optimized and are not ready to be used in real-world applications. Considering this, we agree with the reviewer that the demonstrations in this paper are conceptual. There are many works that need to be done (probably customize the structures and design for specific surgical treatments with optimization in materials, fabrication process, and surface treatment) before using them for clinical applications.

However, we believe the presented technology holds strong potential for designing new surgical tools that can be reconfigured into different stable shapes. Specifically, we have shown multiple new features of MaSoChains that are difficult to achieve for conventional catheter designs. We respectfully disagree with the reviewers' comments regarding Fig. 4 (for details, see the next paragraph). Considering that this is the first paper proposing this mechanism, at this moment, it is very difficult to fully evaluate the clinical potential of this technology, and we are looking forward to further developing it with our clinical collaborators and customizing them for specific treatment. In this paper, we focus on understanding the fundamental mechanism of this method and demonstrating these new features of MaSoChain in simplified and abstract application scenarios (Figs. 4-7). We hope that researchers in relevant fields can better understand these new features through our demonstrations and can lead this idea further to more realistic and clinically relevant applications.

In the following paragraphs, as reviewer 1 requested, we explained the key new features (in Figs. 4 and 5 and a new Fig. 9) and provided the background context of why it is difficult to realize through conventional technologies. We also rewrote part of the main text to make it clearer for the readers.

In Fig. 4, we show that using a simple folding mechanism, we can expand the access regions of the tip of the MaSoChain. The reviewer noted that one can easily reach the same point of the tip by retracting the catheter (very smart idea!). However, there are two differences:

1. When navigating in the human body, the anatomy imposes constraints on the configuration the tool can take. Here, we use cardiac ablation as an example (Fig. R1). The same analysis can apply to other enclosed champers (e.g., Bladder). To access the internal surfaces of an enclosed chamber, the catheter/MaSoChain needs to first enter the chamber and then bend backward to access the surface points shown in Fig. R1.
2. The contact angles between the catheter tip and the tissue surface are different. For cardiac ablation procedures, the contact angle of the ablation catheter can influence the ablation results since the electric power can be absorbed by the surrounding blood as the temperature increases rather than delivered to the targeted tissue[1,2]. It is common for the surgeon to control the perpendicular contact angle between the ablation catheter tip and the tissue to ensure the best ablation results.

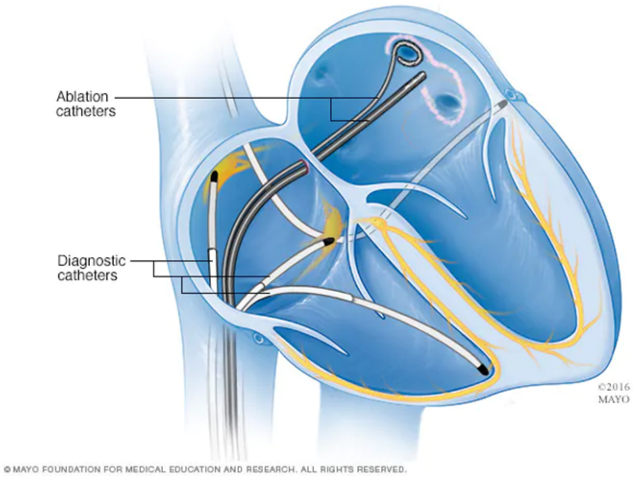

Fig. R1, **Illustration of cardiac ablation using catheters.** The regions close to the access points are always difficult to access since they require the catheters to bend 180 degrees. The image was taken from the Mayo Clinic website ([Cardiac ablation - Mayo Clinic]).

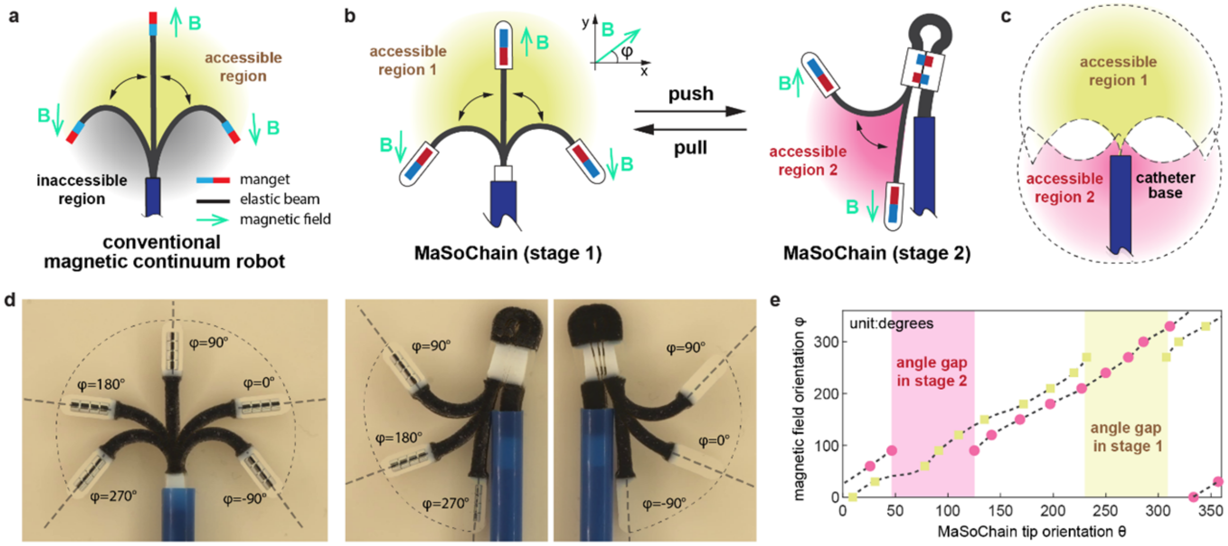

Fig. R2 (the updated Fig. 4) Expanded accessible regions for magnetically guided catheters. **a** A schematic illustration of a conventional magnetic continuum robot. Based on the reachable angles of the tip under a dynamic magnetic field, we marked the surroundings as the "accessible region" (in yellow) and "inaccessible region" (in black). **b** Accessible regions of two stages of the MaSoChain by pushing and pulling relative to the sheathing tube/catheter. **c** Combined accessible regions of the two folding stages, showing complete position and angle coverage around the holding sheathing tube. **d** Experimental results of overlapped images of the catheter position, orientation (dashed straight line), and corresponding magnetic field orientation φ. The external magnetic field is 10 mT rotating inside the same plane. The complete demonstration can be seen in Supplementary Video 3. **e** The complete accessible regions (yellow and red) of both working stages of the MaSoChain, referring to the base tube/catheter. **f** Experimental comparison between the tip angle and the magnetic field angle. Under a 360-degree magnetic field, each stage has a gap that cannot be covered; however, by combining

the two stages, a full angle of 360° is covered and provides more options for two different configurations with the same contact angle.

Based on the above two reasons, we argue that the foldable MaSoChain can provide new opportunities to increase the accessible regions of the catheter tip. The simple structure of just two folding segments can easily expand the accessible region into stage 2 (in Fig. R2) without the need for a large bending torque and avoiding nonlinear mechanics near the singularity[3]. To clarify the difference, we added a new plot in Fig. 4, highlighting the tip angle theta and the corresponding magnetic field direction angle phi, with detailed explanations in the figure caption.

In Fig. 5, we agree with the reviewer that “pick up and place” is a merely conceptual demonstration. The purpose of this demonstration is to show that MaSoChain can assemble into tools that are significantly larger than the diameter of the hosting tube. In this specific case, we chose to fold into a large gripper and demonstrate it in vitro. However, we believe that such an assembly mechanism can be potentially useful in the stomach, especially considering the two examples using the capsule robot:

(1) Battery retraction:

In this paper[4], the authors show that a mobile magnetically controlled foldable robotic capsule can move inside the stomach and actively grab the button battery. Every year, more than 3500 people ingest button batteries in the United States, which can lead to severe esophageal or airway burns and subsequent complications[4].

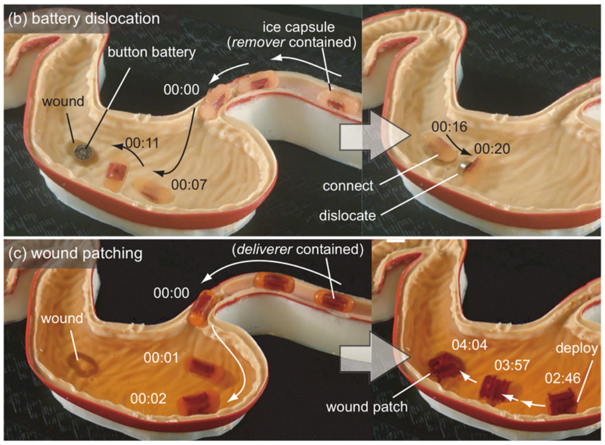

Fig. R3, Battery removal and wound patching experiments. The ice capsule containing the remover thrown manually into the stomach was controlled remotely and connected to the button battery. The ice capsule successfully dislocated the battery from the site. The figure is adapted from reference[4].

Using MaSoChain, it is possible to perform similar experiments through new customized designs based on the specific patient and battery. It is also possible to use a miniaturized version of MaSoChain to remove the battery using magnetic interaction between the NdFeB on the MaSoChain and the battery.

(2) Folded pills for long-term drug therapy

In a recent publication[5], researchers showed that by folding into a larger star-shaped structure, it can stay in the stomach for long-term drug release. However, the geometries are limited by the size of the capsule.

MaSoChain can overcome this limitation by folding some truly large structures delivered through a tube/catheter. This large structure will not only add more space for more drugs but also allow more functionalities, including long-term monitoring with onboard electronics, trigger release/stop using an external electromagnetic field, and complex disease management. We are actively exploring these potentials with our clinical partners in future work.

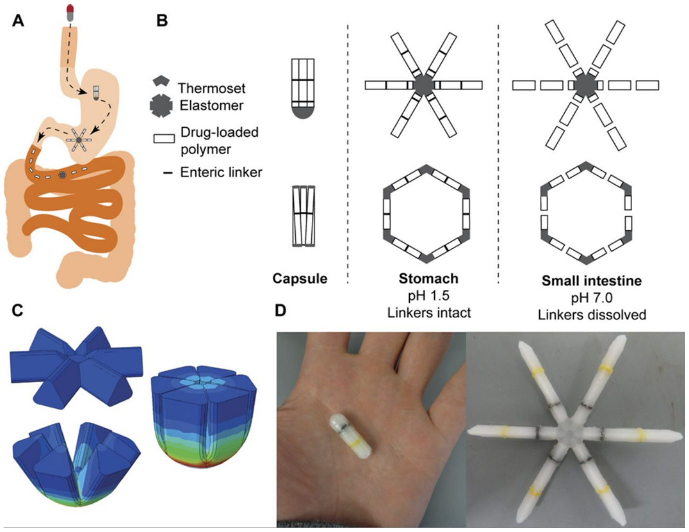

Fig. R4, Design of a modular gastric residence vehicle. Schematic of the deployment of the gastric residence drug delivery dosage form via an ingestible capsule. The image is adapted from reference[5].

Responding to the reviewer's comment, we revised our paper to demonstrate our concept for more realistic applications. We added a new Figure 9 demonstrating a self-folding capsule endoscope based on MaSoChains. The reconfigurable system is composed of three functional modules: a camera module, a large NdFeB steering magnet that can be driven by an external magnetic field, and a working channel allowing a biopsy gripper to pass through.

This self-folding capsule endoscope has addressed an important limitation of the current endoscope designs in that all the functional components need to be integrated at the tip of the endoscope. This geometric limitation prevents further miniaturization of the current endoscope. We think the self-folding structure can address this issue. With MaSoChain, we can distribute the functional component along MaSoChain and fold it into the desired geometry when pushing out of the tip in an open space (e.g., stomach). We have demonstrated the basic operations of this capsule endoscope in Fig. 9, Supplementary Movie 9, and more discussion in the new section "Demonstration of a self-folding capsule endoscope". Although the size we demonstrated is still quite large, we believe the concept can facilitate further miniaturization for many existing endoscopic devices. MaSoChains can provide unique advantages in navigating

inside the GI tract (e.g., passing through a small channel) and reduce pain due to tissue stretching[6].

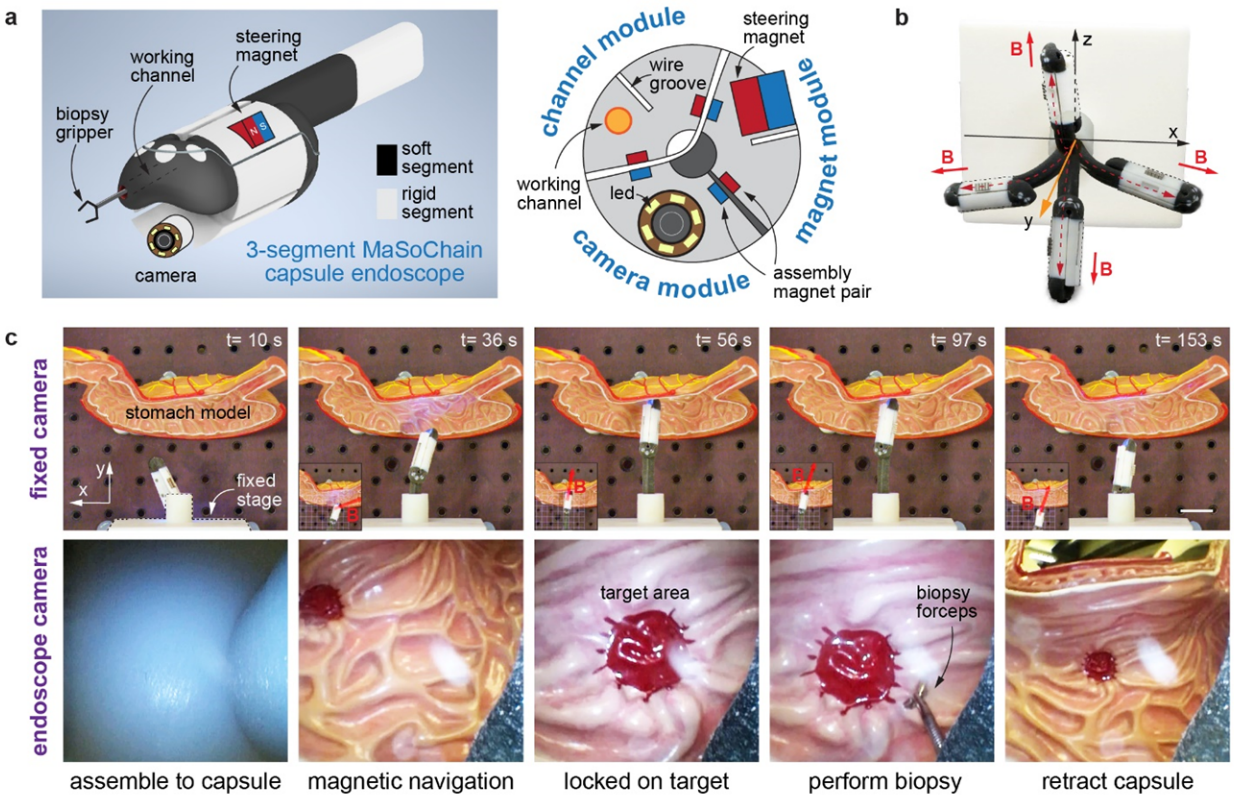

Fig. R5 (the new Fig. 9) A foldable endoscope demonstration using MaSoChain. **a** Structural design of a foldable endoscope with 3 functional segments: the camera module, the magnet module with a large steering magnet and the channel module through which a biopsy gripper can pass. The three-segment MaSoChain structure can self-assemble into a capsule endoscope when pushing out the tube. **b** The capsule endoscope can be actively steered using an external magnetic field. The capsule position and orientation are shown with corresponding magnetic field directions. **c** Demonstration of the use of the MaSoChain endoscope with both the fixed lab camera view (upper row) and the onboard endoscope camera view (lower row). The scale bar is 25 mm. The process showed a complete assembly of the three-segment capsule endoscope, magnetic navigation of the capsule, locking on the target, insertion of biopsy forceps, performing biopsy, and retraction of the capsule. The complete experiment can be found in Supplementary Movie 9.

The overall goal of this paper is to provide a complete understanding and characterization of the folding mechanism of MaSoChain, with our best effort to demonstrate the potential of the technology.

1. The whole claim of this paper is based on the assumption that the MaSoChain is compatible with a catheter, not a plastic tube. A catheter first delivers the MaSoChain to the target position, and the MasoChain is then reconfigured into a larger shape. If MaSoChain is not compatible

with the commercial catheters, the MaSoChain cannot even be delivered to the target position, and the practicality of this concept is not guaranteed. However, it appears to me that the author did not conduct the experiment with a commercial catheter. In particular, the supporting sheath in Fig. 5 appears to be a very stiff catheter or a plastic tube, and the MaSoChain is moved from one position to another by literally moving the whole tube with a human hand which is infeasible in realistic cases. Is this MaSoChain compatible with a commercial catheter?

In this work, we did not differentiate catheters and tubes because they both share tubular geometries. What we understand is that catheters are made of medical-grade materials and are developed for specific applications. Depending on the applications, the structures can be slightly modified, including different tip cuts, bends with different angles, and distributed holes, for better liquid suction performance.

We agree that medical-grade catheters are different from the rigid tubes used in our original demonstrations. To compare the differences, we try to provide a list of key differences, and we consider their effect on the folding/unfolding process of the MaSoChain. We also purchased medical grade thoracic catheters (Covidien thoracic catheter, PVC, 24FR, straight, inner diameter 6 mm, sterile) and used them as a reference to perform tests with our 3D-printed MaSoChains.

(1) The stiffness of the tube (or modulus of the materials)

    Many materials have been used for making medical-grade catheters (polyethylene, polypropylene, polyurethane, polycarbonate, polyetherimide, Pebax, Nylons, etc.). For most catheters, the tube walls are sufficiently thick to prevent local large deformation but still allow bending in the radial direction. As a result, we did not observe any difficulties in the push and pull of MaSoChain relative to the sheathing catheters.

(2) The curvature of the catheter

    This is a very important parameter since it limits the length of the rigid segment of MaSoChain. If the hosting has a very small local curvature, a long rigid segment of MaSoChain may not pass through.

(3) Tip geometry

    The disassembly of two requires segments to be separated by the tip of the catheter. The tip geometry has a direct impact on the disassembly performance.

(4) Surface coating

    For medical-grade catheters, surface treatments are commonly performed to reduce friction and prevent the adhesion of proteins and other biomolecules. We see that this can only assist the use of MaSoChain. We believe that MaSoChain needs to perform a similar coating process during further optimization for in vivo experiments.

In fact, in many medical applications, medical catheters are highly customized for specific procedures. As we found, some companies, such as https://www.creganna.com/technologies/, provide outsourced design and manufacturing solutions for minimally invasive delivery surgeries. We also found a useful introduction article that shows the state-of-the-art catheter design and manufacturing from a company named Interplex, which also provides consulting and

customization services. (link to the article: Catheter Design and Manufacturing: All about the Technology Behind it (interplex.com))

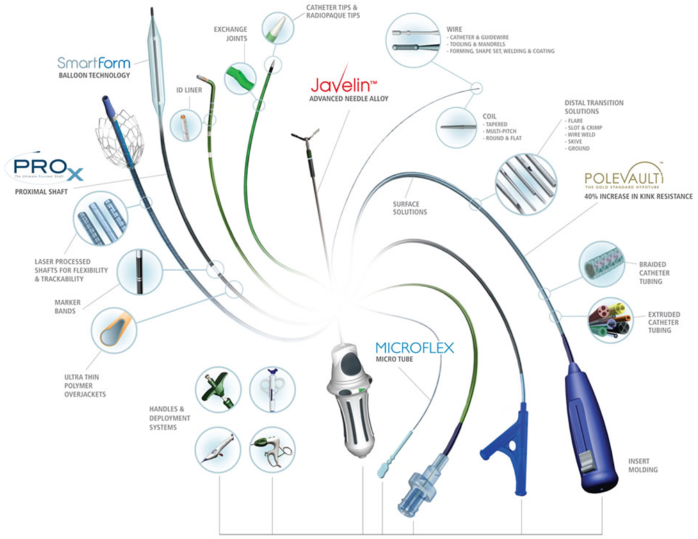

Fig. R6, Customized catheter design and manufacturing provided by Creganna Medicals. The image is taken directly from the website: https://www.creganna.com/technologies/.

Based on that information, we have reasons to believe that depending on the different implementations of the MaSoChain (different sizes and different functions), the hosting catheters/tubes will be quite different from each other and would likely be customized and packaged together with the MaSoChain as a medical device if this technology makes its way to the market. Therefore, we do not consider the compatibility issue as a major challenge for the proposed MaSoChain technology.

To address the reviewer's concern, we also performed experiments with commercially available medical-grade theoretical catheters (Covidien thoracic catheter, PVC, 24FR, straight, inner diameter 6 mm, sterile). Based on our experiences, we did not find major differences between rigid tubes and medical-grade catheters in straight catheters with flat tips. We used medical-grade thoracic catheters for the experimental force measurement of assembly and disassembly tests to provide more information. The detailed force testing results can be found in the next question and question i) from the second reviewer.

2. The pulling process of this MaSoChain will apply some amount of force on the tip of the catheter. The force between the two coupled magnets might be enough to flatten the tip of the catheter, which is undesirable consequences. The deformed catheter can block the deployment of other devices and the sharpened tip might leave scratches on the surface of organs. Does the force between the magnets cause any deformation on the tip of the commercial-grade catheters?

This is a very good question. The force involved in the disassembly process is not systematically studied in the initial submission, and we share the same concerns. As reviewer 2 also suggested, we investigated the magnetic force between the MaSoChain segments during the disassembly process through both numerical simulations of magnetic interactions and experiments using a customized force testing setup. Afterward, we will evaluate the risk of plastic deformation and permanent damage to the tip of the catheter due to the disassembly of MaSoChain. The catheters used for the experiments are actual medical-grade thoracic catheters (Covidien thoracic catheter, PVC, 24FR, straight, inner diameter 6 mm, sterile) selected based on a size comparable to that of the MaSoChains fabricated by our multimaterial 3D printer.

Modeling of the magnetic force between two NdFeB assembly magnets

The two assembly magnets are in close contact with each other during the assembly and disassembly process. The geometry of the assembly magnets needs to be considered for force estimation. This means that we cannot use a simplified point-based dipole–dipole interaction model for the magnetic interactions because they rely on the assumption that the distance between two dipoles is much larger than the magnet size.

To include the effect of the geometries, we wrote a MATLAB script to calculate the interactions between the two assembly magnets (block NdFeB: 1.2×1×0.5 mm, dipole direction is along the 0.5 mm axis) based on the finite element method, where we consider each assembly as a small number ($o \times p \times q$) of unit magnets at different locations. Then, we can represent the interaction between the two large assembly magnets as a summation of dipole–dipole interactions between each small magnet pair at its own geometric center (Fig. R7). This method can effectively include the geometric factor of the assembly magnets, providing an accurate estimation of force in the disassembly process. More details can be found in the Methods section: Modeling of the magnetic force between two NdFeB assembly magnets.

Magnetic dipole–dipole force:

$$\mathbf{F}_{m_1-m_2} = \frac{3\mu_0}{4\pi|\mathbf{r}|^4}\{(c \times \mathbf{m}_1) \times \mathbf{m}_2 + (\hat{\mathbf{r}} \times \mathbf{m}_2) \times \mathbf{m}_1 - 2\hat{\mathbf{r}}(\mathbf{m}_1 \cdot \mathbf{m}_2) + 5\hat{\mathbf{r}}[(\hat{\mathbf{r}} \times \mathbf{m}_1) \cdot (\hat{\mathbf{r}} \times \mathbf{m}_2)]\}$$

Magnetic energy potential between two point dipoles:

$$H_{m_1-m_2} = -\frac{\mu_0}{4\pi|\mathbf{r}|^3}[3(\mathbf{m}_1 \cdot \hat{\mathbf{r}})(\mathbf{m}_2 \cdot \hat{\mathbf{r}}) - \mathbf{m}_1 \cdot \mathbf{m}_2] - \mu_0 \frac{2}{3}\mathbf{m}_1 \cdot \mathbf{m}_2 \delta(\mathbf{r})$$

Total force between two assembly magnets:

$$\mathbf{F}_{total} = \sum_{i=1}^{i} \sum_{j=1}^{j} \mathbf{F}_{m_i-m_j}$$

Total magnetic potential energy between two assembly magnets:

$$H_{total} = \sum_{i=1}^{i} \sum_{j=1}^{j} H_{m_i - m_j}$$

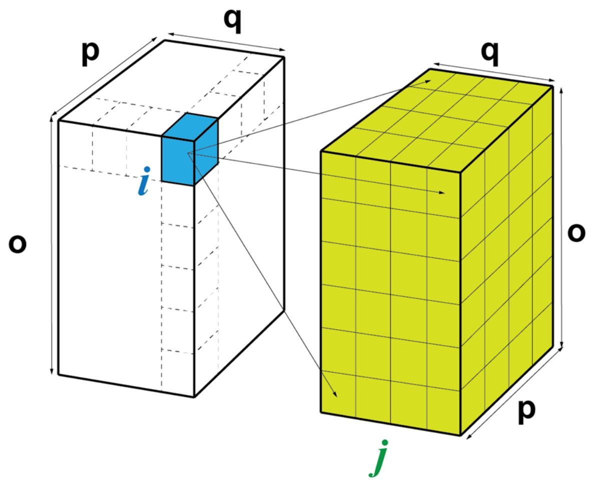

Fig. R7, Illustration of a finite element method to calculate the magnetic force and potential between two close block magnets.

Based on the equation and method mentioned above, we calculated the magnetic force between two assembly magnets (numbers 1 and 2) with different relative positions. As shown in Fig. R8, we fixed the central position of magnet number 2 at position P_2(-0.5,0,0) (unit: mm). We move the central position of magnet number 1 in the workspace and calculate the corresponding magnetic force and energy. P_1(x,y,z) represents the central position of magnet number 1. The scanning volume is marked in Fig. R8, from 0 to 3 mm in the x-direction, -3 to 3 mm in the y-direction and -3 to 3 mm in the z-direction. We also use the same coordinates for plotting the calculated results.

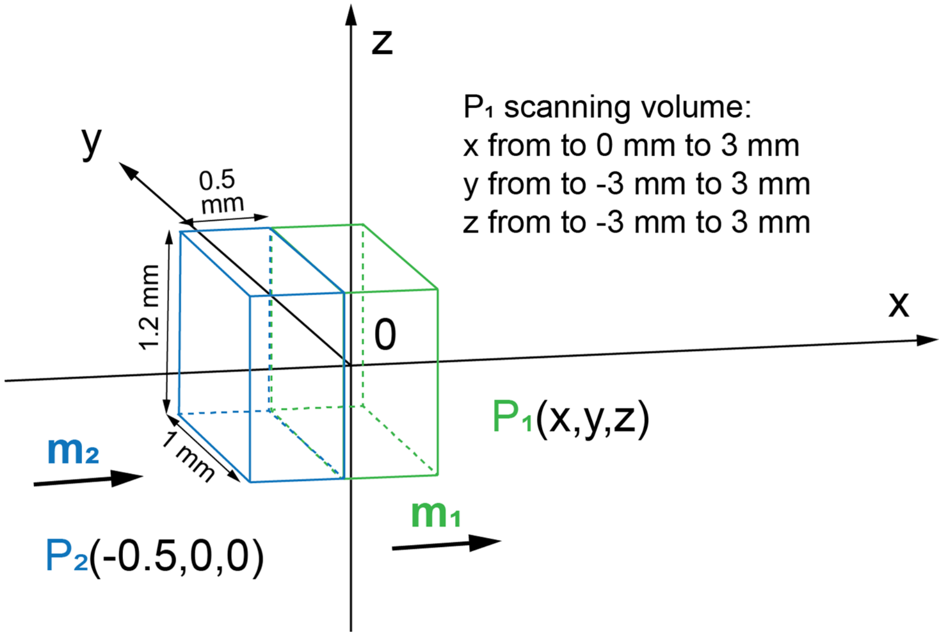

Fig. R8, Illustration of the coordinate system and the relative positions between two assembly magnets. Magnet number 2 is fixed with central position P_2(-0.5,0,0), and P_1(x,y,z) scanning in the workspace.

The calculated magnetic potential energy and assembly force are shown in Fig. R9 and Fig. R10, respectively.

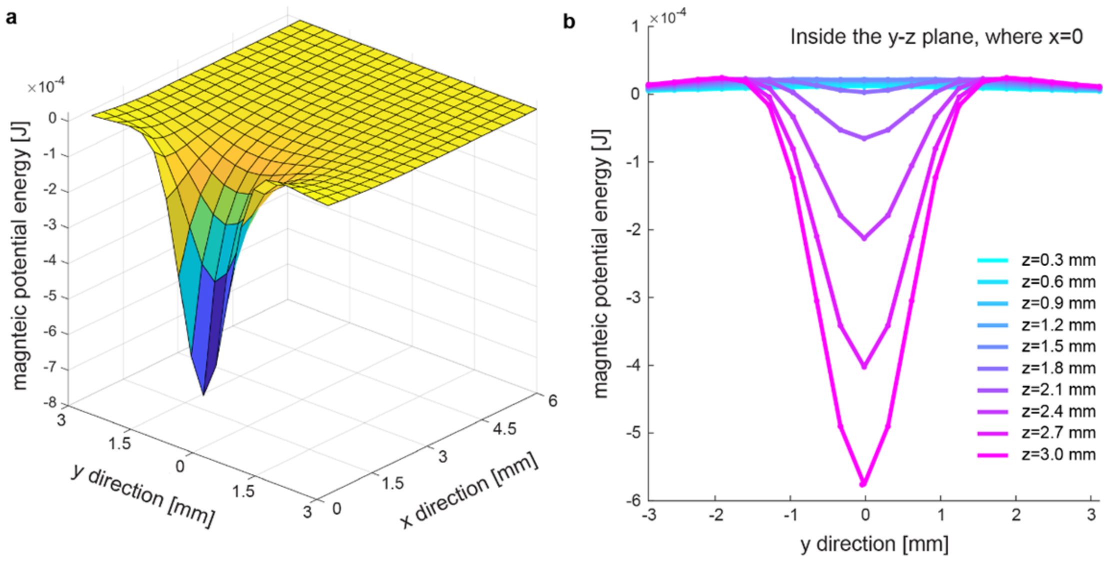

Fig. R9, Calculated magnetic potential between the two NdFeB assembly magnets.

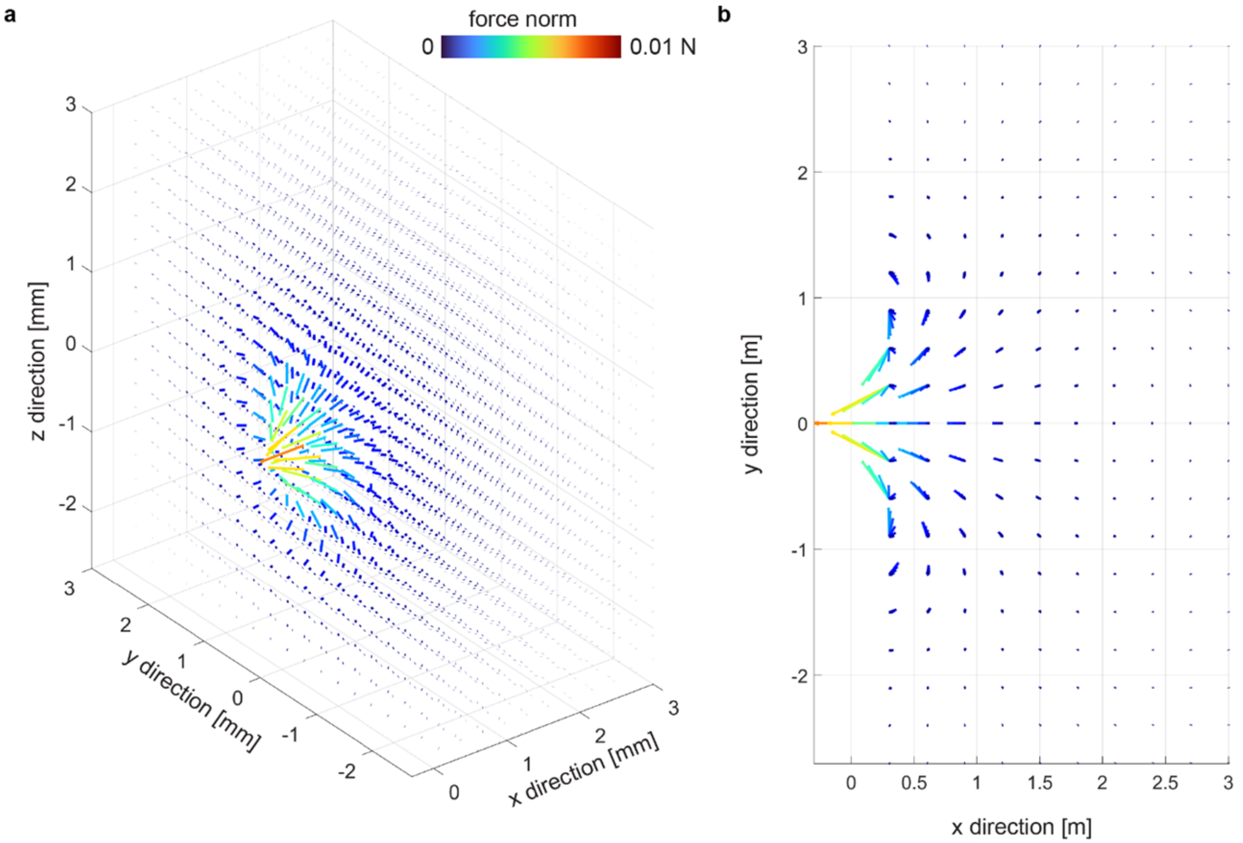

Fig. R10: Simulated assembly force between the assembly pair magnets (two NdFeB magnets with the same dipole direction). The original configuration of the simulated configurations can be found in Fig. R8.

Experiments on the disassembly force of MaSoChain

Based on the numerical simulation results, the disassembly force is approximately 0.01 N. However, the magnet force is only small part. When disassembling the MaSoChain, soft segments are also heavily deformed, and the friction is not negligible. We build a customized force measurement setup to measure the axial force related to the assembly and disassembly process, as shown in Fig. R11. More details can be found in the methods section.

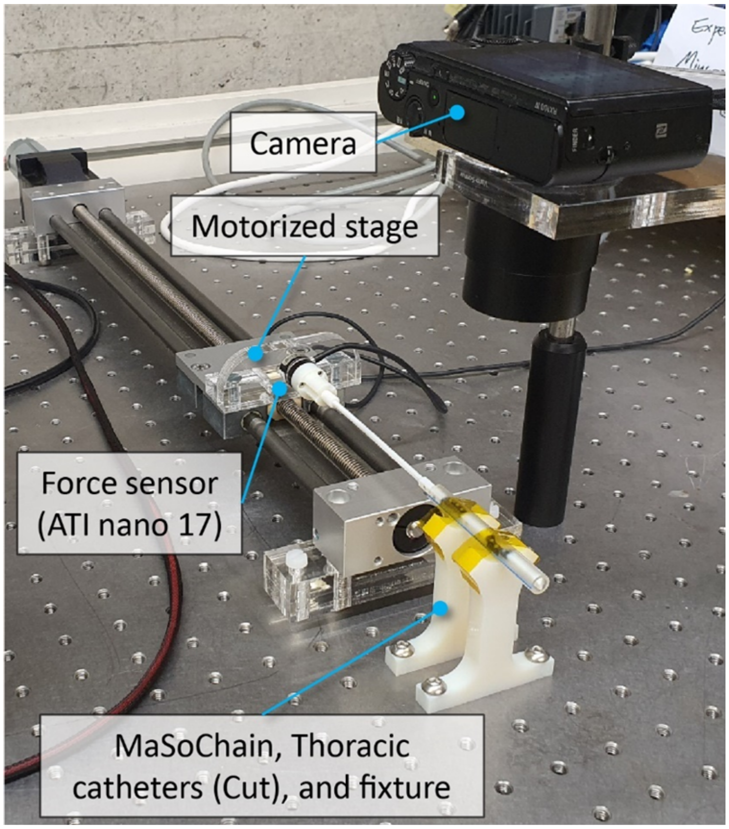

Fig. R11, Experimental force measurement setup. The motion of the MaSoChain is controlled by a motorized stage at a constant speed. The force along the axial direction between the MaSoChain and the thoracic catheters is measured.

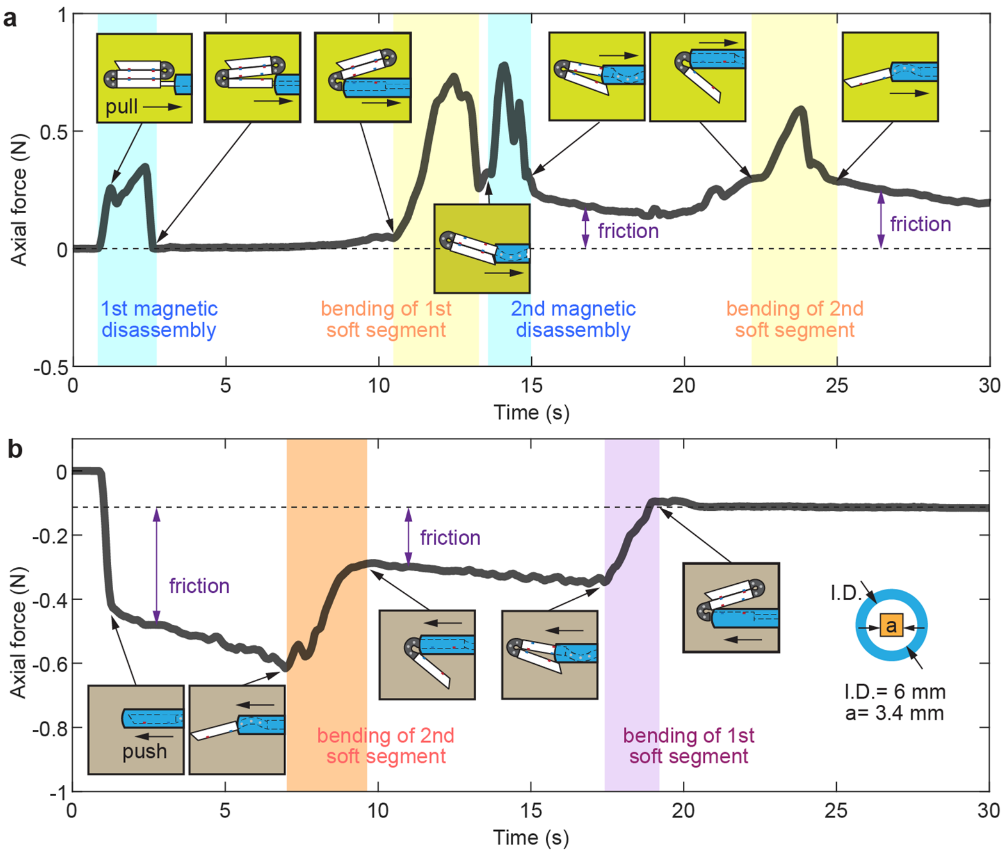

Fig. R12 (new Fig. 8), Force characterization of MaSoChains assembly and disassembly inside a medical grade thoracic catheter. The pulling/pushing force along the axial direction during the disassembly (panel **a**) and assembly (panel **b**) are measured. The measurement rate is 1 kHz, but the curve is smoothed with a moving average of the nearest 250 points. The folding process involved bending of the soft segments and magnetic disassembly. Key moments are marked along the force–displacement curve with small inset diagrams to show the instant shapes of MaSoChains. More information can be seen in Supplementary Movie 8 and the supplementary information.

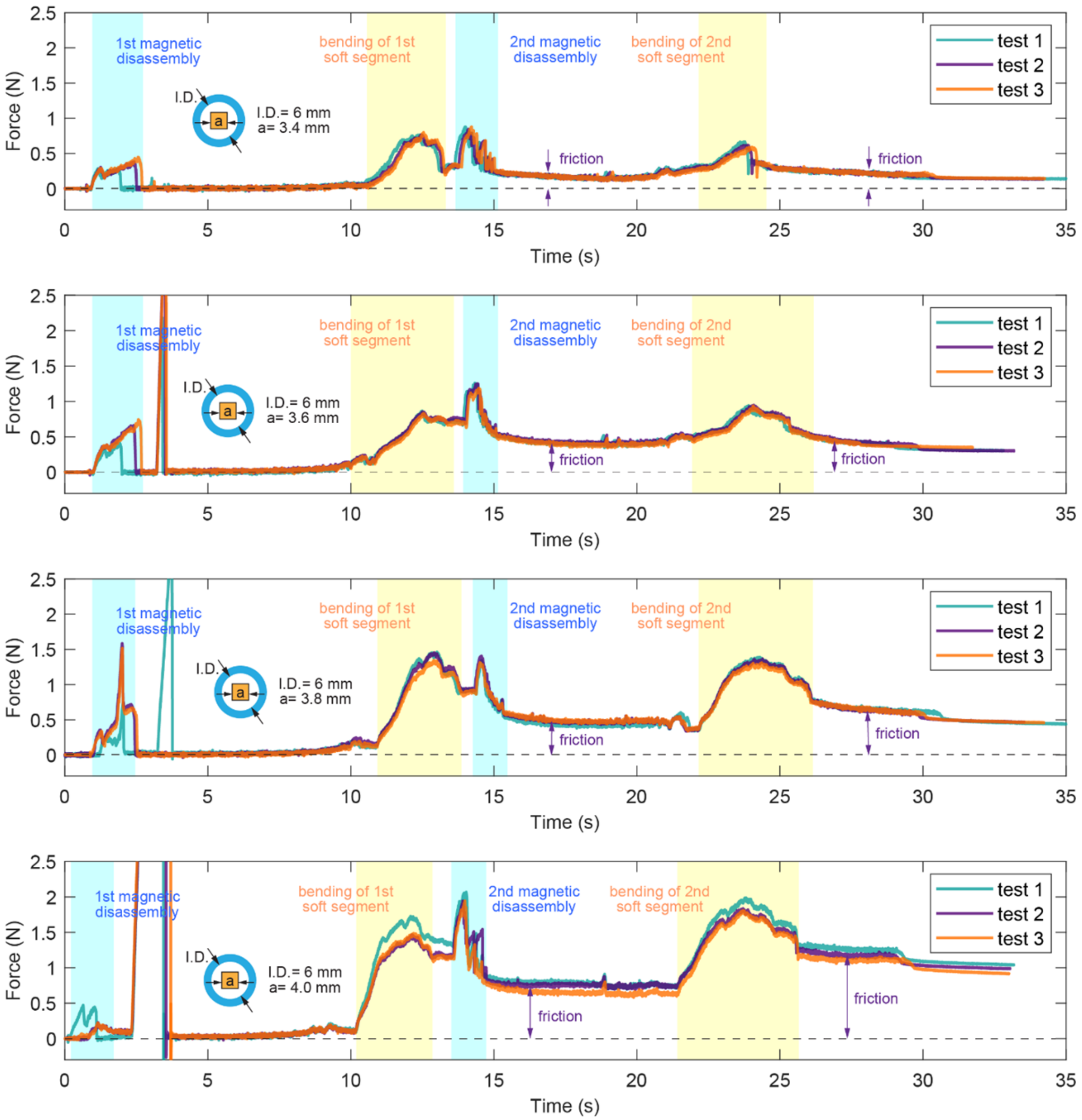

Fig. R13, The complete force measurement results between a three-segment MaSoChain and medical grade thoracic catheter during the disassembly process. Four MaSoChain samples are tested with different cross-section dimensions (square length: 3.4 mm, 3.6 mm, 3.8 mm, and 4.0 mm). The 24 French thoracic catheter has an inner diameter of 6 mm. The pulling force along the axial direction is measured with a 1k sampling rate.

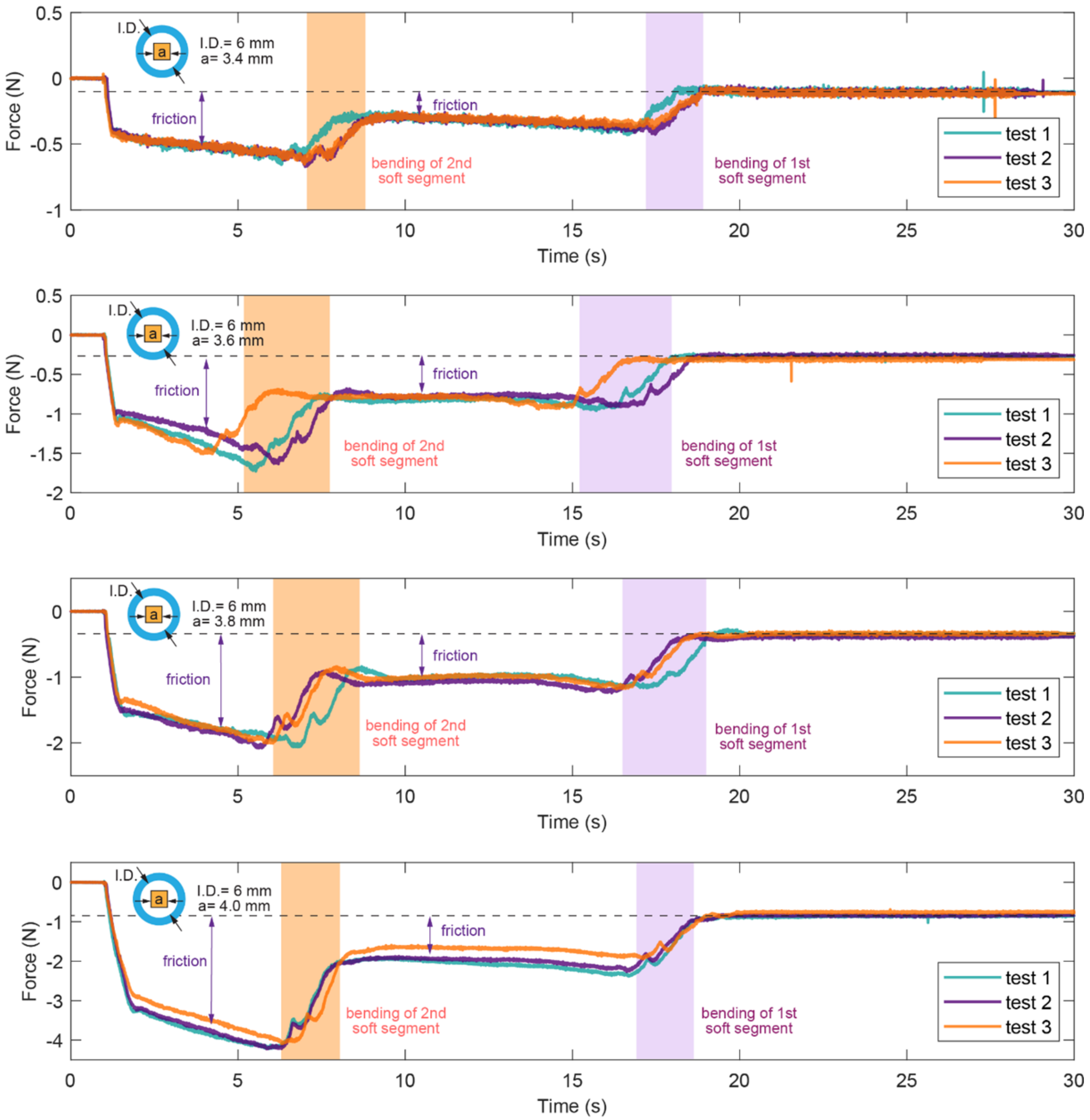

Fig. R14, The complete force measurement results between a three-segment MaSoChain and medical grade thoracic catheter during the assembly process. Four MaSoChain samples are tested with different cross-section dimensions (square length: 3.4 mm, 3.6 mm, 3.8 mm, and 4.0 mm). The 24 French thoracic catheter has an inner diameter of 6 mm. The pushing force along the axial direction is measured with a 1k sampling rate.

The concern that the magnetic force between two assembly magnets can potentially damage the tip of the catheter is very unlikely to happen. Based on our simulation and experiments, the maximum force between the assembly magnets is approximately 0.01 Newton, which is equivalent to a weight of 1 gram. The measured pulling force required for disassembly is less than 1 Newton, which includes the friction, soft segment bending force and magnetic force. The materials (e.g., PVC, silicone, etc.) used for making catheters are typically quite resilient and robust to certain levels of stretching and deformations. Therefore, we can safely conclude that MaSoChain is unlikely to destroy the tips of catheters.

3. Once the soft robot is reconfigured into a larger shape, the soft segment located in the vicinity of the catheter tip must withstand the weight of the deployed tool. Otherwise, the deployed device will be bent toward the direction of gravity, which makes it difficult to control. Does this concentrated weight of the folded structure cause the sag of the tool when it is deployed in 3d space? In addition, can these tools be further advanced from the catheter tip without the sag? This paper only shows cases where the deployed tools are actuated near the tip of a catheter.

This is a very good question. The density of our 3D printed material for the soft segment (Agilus30, Stratasys) is 1.07 g/cm^3 and that of rigid materials (VeroWhitePlus, Stratasys) is 1.1 g/cm^3. The typical weight of a rigid segment is approximately 0.135 grams (3x3x15 mm), corresponding to a bending torque of $0.1\ N \cdot mm$ (weight 0.0135 N times the half-length of 7.5 mm). As a comparison, the elastic torque that bends the assembly structure is $0.5\ N \cdot mm$ (from Fig. 2). This shows that gravity is not truly our top concern for simple structures.

However, as the reviewer mentioned, with an increasing assembly size, the gravity of the overall assembly can become large and may influence the dynamics. To resolve this issue, our suggestion is to increase the elastic spring constant of the soft segments. MaSoChain can also be implemented with other sizes and with different materials for ideal performance. For example, for our large-size implementation with flexible electronics (cross-section: 5x5 mm) and the last demonstration of a foldable endoscope, we observed much less bending under gravity due to the higher stiffness of the soft segment. However, this trend is not in our favor due to the currently limited choice of the stiffness of 3D printing materials. We also provide a possible solution to this for miniaturization to submillimeter scales as our answer to the 2nd question from reviewer 3.

We envision that MaSoChain should have a stiffness similar to that of existing medical catheters/guidewires to provide both flexibility and position accuracy. This requires one to optimize the design based on the number of segments and size of the overall assembly of the MaSoChains. For MaSoChains, which is very small (as shown in Fig. 4), for potential cardiac ablation catheters, one can implement a similar structure. For much larger assemblies with tens of hundreds of segments, we think it is crucial to improve the fabrication strategy to provide a much higher elastic modulus to prevent unwanted bending due to gravity. We are also looking forward to exploring those opportunities in different applications in future work.

Reviewer #2 (Remarks to the Author):

The paper presents a novel solution for assembling large 2D and 3D structures inside the body cavities thanks to self-folding magnets. The idea behind the paper is clever and the fabrication modalities are accessible. In addition, the videos are very interesting and well explain the obtained results.

The reviewer is very positive towards the paper.

On the other hand, the reviewer thinks that the paper could be improved with i) some more details about the pulling forces needed to disassembly to structure; ii) a retuning of some references of the state of the art; iii) a clearer explanation of the effect of external magnetic fields and of the extra magnets.

We are very grateful for the very positive evaluation and insightful suggestions. We have performed extra modeling, experiments, and analyses to address all your questions.

More details are reported below:

i) The delivery of the structures from the catheter should be straightforward, even if the speed of delivery is not analysed. The reviewer thinks that the assembly could be less or more effective depending on the speed of delivery.

This is a very insightful comment. We are impressed by the reviewers' observations and instinctive guesses. Indeed, the reviewer is correct about the assembly depending on the insertion speed.

The reason for this dependence on the insertion speed is mostly due to the viscoelastic nature of the 3D-printed soft elastic segment (black part as in Fig. 1). This means that the soft segment, once released from the sheathing tube, will need slightly more time to fully bend and assemble to the targeted geometries until the magnets click. This phenomenon is clearly recorded in Supplementary Video 3. The time required for folding can vary with the cross-section size, bending angle, segment length, and resistance forces in the assembly process. In our experiments, we always wait sufficient time for the segments to be fully assembled. Based on our observations, the typical time constant for the 3x3 mm cross-section segment is approximately 3 seconds, and the typical assembly time for the 5x5 mm cross-section segment is approximately 2 seconds.

However, the observed "long" folding time is not our intention but due to the viscoelastic effect in the 3D-printed soft materials, which is an intrinsic limitation of our 3D printer. Recently, the mechanical properties of 3D-printed materials have rapidly improved, and we hope this challenge can be solved with new printers. MaSoChain can also be fabricated using other methods, including using medical-grade superelastic nitinol. We shared our vision for potential methods to fabricate miniaturized MaSoChains in response to the 2nd question of reviewer 3.

Anyhow the most relevant issue is the retrieve of the structure: which are the forces necessary for pulling back the structure and disassemble it? Which is the risk of getting stuck at a certain point at the tip?

This is a very important question. The first reviewer also expressed similar concerns. In the revised manuscript, we provide a systematic study through both numerical simulation and experiments. Please find detailed responses to question #2 of the first reviewer.

Can an external magnetic field help to disassembly the structure in case of problems (e.g. by producing a sort of opposite field on to the small magnets)?

Our design is made so that an external magnetic field will not influence the assembly. On each rigid segment of MaSoChain, we intentionally arrange two assembly magnets with opposite dipole directions. This specific design allows us to eliminate the magnetic torque on the assembly magnets. In a uniform magnetic field (which is a valid assumption for most medically relevant magnetic actuation and navigation systems), the assembly magnet pair always has an opposite dipole moment, resulting in zero magnetic torque regardless of the external magnetic field direction. This design allows us to decouple the assembly magnets with “torque magnets”, which are added to provide a strong magnetic actuation torque in Fig. 4 and Fig. 9. In other cases where a strong magnetic field gradient is applied, we also provide detailed analysis in the response to question (iii) regarding the assembly stability near a large permanent magnet.

In addition, friction during the structure delivery and pulling could be relevant, but the friction problem is never mentioned, except in the last lines (“In the future, one can implement commercially available superelastic nitinol alloy to overcome these limitations and reduce the friction with the sheathing catheter”).

This is another very good question. In many minimally invasive procedures, the friction between the catheter tube and other instruments (e.g., guidewire, biopsy gripper, etc.) is important, and there are many methods to reduce friction that are implemented for commercially available medical devices (e.g., surface coating). MaSoChain requires a prebent hybrid chain composed of rigid and soft materials to pass through a confined channel, and the internal torque, which is balanced by the normal forces on the internal surface of the catheter tube, generates extra friction force. To address the reviewer’s comment, we performed systematic experimental studies on the friction force between MaSoChain and a medical-grade thoracic catheter (Covidien thoracic catheter, PVC, 24FR, straight, inner diameter 6 mm, sterile).

In Figures R13 and R14, we measured the pulling and pushing forces during the assembly and disassembly process. In those figures, we can have a hint of the friction level between the MaSoChain and the thoracic catheter. In the figures, there are regimes in which the force drastically changes due to the disassembly of the magnets and shape change of the soft segment of MaSoChain, which are marked with color blocks on the figure. Between those regimes, the relationship between shape transformation and the pulling force is rather constant, and we did not observe important geometric changes. We think this force level can represent the friction level between the masochain and the thoracic catheters. We marked this level of

force as friction. We notice that this “friction” force changes with the number of contacted segments of MaSoChain inside tube, and it also changes with the size of the masochain with respect to the catheter inner diameter.

We note that the material is directly printed by our 3D printer without any surface treatment. We think the future versions of the devices made into clinical applications can be performed by a qualified company with customized lubricative materials with proper surface treatment.

ii) When the authors mention the state of the art of reconfigurable robots for surgery and intervention, some seminal works on magnetic retractors, magnetic frames, magnetic cameras could be mentioned. In those cases the access for the magnets is from the mouth or from the abdominal trocar. So the size is larger, but the concept is not far from what the authors are presenting here (e.g. see G. Tortora, 2014, IEEE ASME TMECH).

This is a very good suggestion. Indeed, the idea is very similar; we are very happy to learn this work and add it as a reference. As the author suggested, we also added the following paper as a reference:

Park, Sangtae, et al. "Trocar-less instrumentation for laparoscopy: magnetic positioning of intra-abdominal camera and retractor." Annals of surgery 245.3 (2007): 379.

iii) While the part related to the pure assembly by magnetic forces and elastic energy is clear, when the authors describe the bending and locomotion based on magnetic fields applied externally, the paper leaves some open issues and generates doubts. Is there any risk that the extra bar magnet introduces instabilities in the assembly phase? Are some constraints to be respected in the positioning of the extra magnets to limit instability and/or the generation of secondary structures?

We are grateful for this insightful comment. In addition, this is an excellent question! We share those concerns that motivate us to provide detailed analysis regarding the stability of the MaSoChain assemblies when the assembly pair is near a large permanent magnet (with a strong local gradient). To address this concern, we provide an analytical model to show that disassembling an assembly pair using a large external magnet is very unlikely. We first consider a simple case, and then we generalize the case for more complex configurations.

In the simple case, we fix one NdFeB assembly magnet (1.2×1×0.5 mm) where its center position is at the origin of the Cartesian coordinate system (x=y=z=0). Its dipole position points toward the positive x direction ($\mathbf{x}_1 = [0,0,0]^T$ with magnetic dipole moment $\mathbf{m}_1 = [m_1, 0,0]^T$). Now, we consider placing a dipole magnet on the left in the x coordinate (position: $\mathbf{x}_2 = [-L, 0,0]^T$, with magnetic dipole moment $\mathbf{m}_2 = [m_2, 0,0]^T$), as shown in Fig. R15.

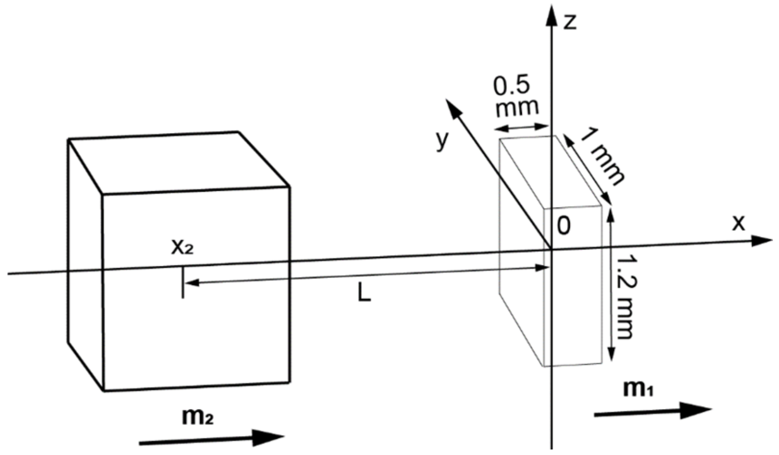

Fig. R15: The magnetic interaction between an assembly magnet $\mathbf{m}_1$ and an external cube magnet $\mathbf{m}_2$.

The magnetic force between the two dipole magnets is:

$$\mathbf{F}_{m_1-m_2} = \frac{3\mu_0}{4\pi|\mathbf{r}|^4}\{(\hat{\mathbf{r}} \times \mathbf{m}_1) \times \mathbf{m}_2 + (\hat{\mathbf{r}} \times \mathbf{m}_2) \times \mathbf{m}_1 - 2\hat{\mathbf{r}}(\mathbf{m}_1 \cdot \mathbf{m}_2) + 5\hat{\mathbf{r}}[(\hat{\mathbf{r}} \times \mathbf{m}_1) \cdot (\hat{\mathbf{r}} \times \mathbf{m}_2)]\}$$

where μ_0 is the magnetic permeability in a vacuum and $\mathbf{r}$ is the relative position vector between the centers of the two dipole moments $\mathbf{m}_1$ and $\mathbf{m}_2$.

$$\mathbf{r} = \mathbf{x}_1 - \mathbf{x}_2 = [L, 0,0]^{\mathrm{T}}$$

After plugging in all the relevant values, the force between two magnets can be expressed as

$$\mathbf{F}_{m_1-m_2} = \frac{3\mu_0}{4\pi|\mathbf{r}|^4} m_1 m_2 \begin{bmatrix} 1 \\ 0 \\ 0 \end{bmatrix}$$

Now let us consider that there are two magnets with opposite dipole directions, as shown in Fig. R16. In this case, two magnets ($\mathbf{m}_2$ and $\mathbf{m}_3$) generate opposite force directions on magnet $\mathbf{m}_1$. We would like to use this case to find the scaling effect of magnet $\mathbf{m}_3$ that can destabilize the assembly pair $\mathbf{m}_1$ and $\mathbf{m}_2$.

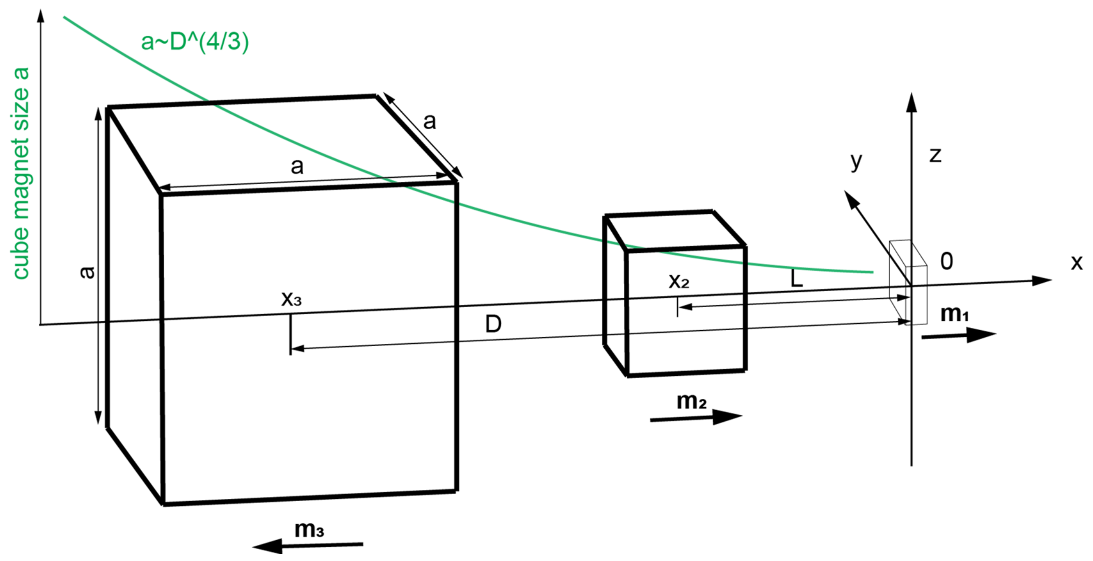

Fig. R16: Force analysis of two competing magnets with opposite dipole directions at different distances.

If we consider both m_1 and m_2 to be NdFeB assembly magnets (1.2×1×0.5 mm), then we consider the third magnet with an opposite dipole direction with magnetic moment $\mathbf{m}_3 = [-m_3, 0,0]^T$, which provides a repulsion force. If we consider that m_3 is sufficiently large that the force can balance the attraction force for m_2, it needs to be balanced; as a result:

$$\mathbf{F}_{m_1-m_2} + \mathbf{F}_{m_1-m_3} = 0$$

$$\frac{3\mu_0}{4\pi L^4} m_1 m_2 - \frac{3\mu_0}{4\pi D^4} m_1 m_3 = 0$$

The required m_3 needs to be large as

$$m_3 = \frac{D^4}{L^4} m_2$$

at position $\mathbf{x}_3 = [-D, 0,0]^T$ to balance the magnetic force generated by m_2. Now, if we assume that m_3 is a cube magnet with an edge size of a, we can rewrite the equation as

$$M_{NdFeB} a^3 = \frac{D^4}{L^4} m_2$$

where M_{NdFeB} is the magnetization of the NdFeB magnet, which is equal to $1.08 \times 10^6\ A/m$. If we now consider D as a variable and consider how a needs to scale with D, we will find

$$a = \sqrt[3]{\frac{m_2}{M_{NdFeB} L^4}} D^{\frac{4}{3}}$$

It shows that with an increasing distance D, the third magnet needs to increase rapidly in size ($\sim D^{\frac{4}{3}}$) to match the force. This means that the magnet needs to be larger than a to destabilize the assembly pair between m_1 and m_2. If the m_2 magnet is touching m_1, as in the assembly pair, the required size of m_3 can be so large that it is physically impossible to fit on the left side. If one changes the m_3 direction, it will only decrease the magnetic repulsion force. The above

scaling law provides an important insight that the magnetic gradient generated by a nearby permanent magnet is very unlikely to destabilize the magnetic assembly pair. The analysis results also resonate with our experimental observations. Therefore, we can conclude that the assembly will be stable in our envisioned applications regardless of the neighboring NdFeB magnet configurations.

Reviewer #3 (Remarks to the Author):

The manuscript by Hongri Gu et al. describes semisoft inkjet 3D printed structures consisting of elastomeric hinges and rigid material parts with an average cross section of strands approximately 10 x 10 mm2. These structures were equipped with locking NdFeB magnets within the rigid parts and flexible printed circuit boards carrying LEDs and heater. The fabricated 3D structures were unwrapped into a chain and inserted into a tubular sheath for further deployment. The structures were deployed by pushing them gradually out of the sheath. Such structures regain the original 3D shape during the deployment process due to the stress stored in the elastomeric parts, while the integrated magnets lock the rigid parts in their correct place preserving the original 3D shape. The integrated flexible electronic circuits bring additional benefits to the structure.

The work has a certain level of novelty and can be potentially interesting for a broad readership. The paper can be accepted after the following points have been addressed:

We are very thankful for acknowledging the novelty of this work and its potential to be accepted for publication. We have carefully answered your comments and remarks as follows:

1) To my knowledge at least one similar approach of magnetic 3D assembly from single strands have been already envisioned previously in e.g. https://doi.org/10.1073/pnas.1910332116 and need to be properly cited.

Thank you so much for this suggestion. The paper is directly relevant to this work, and we added this citation to the revised manuscript.

2) The Authors discuss miniaturization of the fabricated devices several times within the manuscript, often telling that the strategy in not limited by size and comparing their structures with superplastic preformed nitinol medical tools. It would be beneficial for the broad readership to understand how actually one can achieve miniature (cross-sections < 1 mm) self-folding chains offering shape recovery, magnetic locking and integrated electronic circuits? What kind of methods, materials and technologies could be potentially explored to achieve this? The reader expects to see at least a sketch and some discussions on this matter.

This is actually a very good question. We are very happy that the reviewer agrees with us that the concept and mechanism of MaSoChain can be implemented in different sizes and geometries. However, fabrication methods are clearly limited by size. We are happy to explain more details about the different fabrication methods that can be used to implement a folding strategy similar to that of MaSoChain at smaller scales. As the reviewer pointed out, miniaturization of the MaSoChain can potentially open many interesting application scenarios for minimally invasive interventions, especially at the submillimeter scale.

In this work, we used a 3D printer (Connex, Stratasys), which has good printing resolution and can seamlessly integrate multiple materials together. However, the selection of materials from this printer is limited. The soft material (Agilus, Stratasys) that can survive large deformation is too soft for miniaturized MaSoChain, and our implementation of the 3x3 mm cross-section MaSoChain (Fig. 1-4, and supplementary video 1-4) is almost at the limit for the required elastic energy. For smaller MaSoChains, we need to use soft materials with a much higher Young's modulus and a similar maximum elongation rate.

One possibility is to use other 3D printable materials. For example, the durable and tough resin from FormLabs can be a good candidate. Compared to Agilus (Young's modulus: 2.4 − 3.1 MPa), the durable resin has a tensile modulus of 1.0 GPa with a maximum elongation of 55% postcure (reference file: https://formlabs-media.formlabs.com/datasheets/1801084-TDS-ENUS-0P.pdf). The material properties are quite similar to those of PVC (Young's modulus 0.9-1.2 GPa), which is commonly used for medical catheters. We think durable resin will provide sufficient elastic energy for submillimeter MaSoChains with a diameter from 0.5 to 1 mm considering the printing resolution. However, the FormLabs 3D printer can only print one material and may require assembly afterward.

Another possibility is to use metals. Superelastic nitinol (nickel and titanium alloy), which has a typical modulus of 41-75 GPa, can provide very high elastic force and torque at small scales. The superior mechanical performance and excellent biocompatibility make it a popular choice for vascular stents and other precision medical instruments. Nitinol can be fabricated through femtosecond laser cutting, providing the possibility to customize for specific geometries. As requested by the reviewer, we proposed a process to fabricate nitinol-based MaSoChain, as shown in Fig. R17.

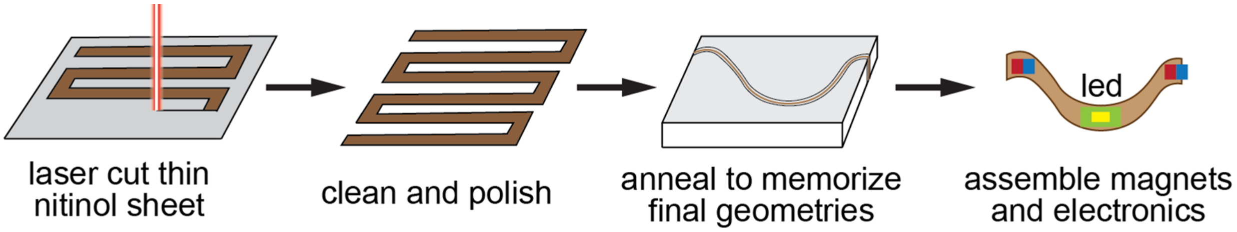

Fig. R17, A potential method to miniaturize MaSoChain to the submillimeter scale using nitinol.

In this proposed fabrication process, we first design and use a femtosecond laser to cut customized stripes from a nitinol thin film. The film is then cleaned, polished and put into a customized mold for the final folded geometry, and the metal mold and nitinol stripe are then placed in the oven for annealing. After cooling to room temperature, electronics and magnets

are assembled onto the MaSoChain, and some coating may be needed to protect the electronics. We think this method might be useful for fabricating MaSoChains with a diameter smaller than 0.5 mm.

The electronics also need to be miniaturized to adapt to the MaSoChain size. This makes conventional flexible PCBs unsuitable for those applications. Fortunately, there are rapid developments in flexible electronics that can be precisely assembled into microfibers[7] or on the surface of balloon catheters[8]. Micromagnet fabrication is also possible with electroplating into precisely shaped micromolds[9,10].

3) Shape sensing with LEDs is not completely clear. First of all, a better schematic and the circuit diagram is required explaining this dynamic process. To my opinion the LEDs can indicate only a few locked states but cannot provide a continuous shape sensing function in the broad meaning, which would be of a great importance for the envisioned application scenario. Please clarify how this approach should assist a surgeon in shape sensing when there is no optical observation feasible, for instance during invasive interventions. Please expand your discussion providing alternative solutions.

We completely agree with the reviewer's comment about the limitation of MaSoChain in Fig. 6. The "on-and-off" signal can only indicate a few assembly states but cannot provide a continuous shape sensing capability of the geometry between those states. In the main text, we only claim: "We also demonstrated a shape sensing structure where the LEDs light up only when it is successfully assembled." As well as "As a result, the light is an indicator of the current state of the geometry of the MaSoChain assembly (Fig. 6h and Supplementary Video 6)."

It would be interesting to consider a MaSoChain with continuous shape-sensing capabilities. For example, one can integrate onboard light sensors to detect light, and the intensity of the light signal can be used as a parameter to evaluate the relative distance/angle between the LED and the sensor. One can also provide multiple degrees of freedom by implementing LEDs with different colors. Other sensor technologies can also be used for local deformation detection. For example, one can use a flexible strain sensor on the surface of the flexible segment and detect the deformation signal based on the change in electrical resistance[11].

It is also important to note that there are many promising technologies for shape-sensing catheters. For example, methods based on fiber Bragg gratings can provide real-time shape sensing of 3D configurations (https://fbgs.com/solutions/shape-sensing/). It is not clear if the proposed MaSoChain can provide better performance.

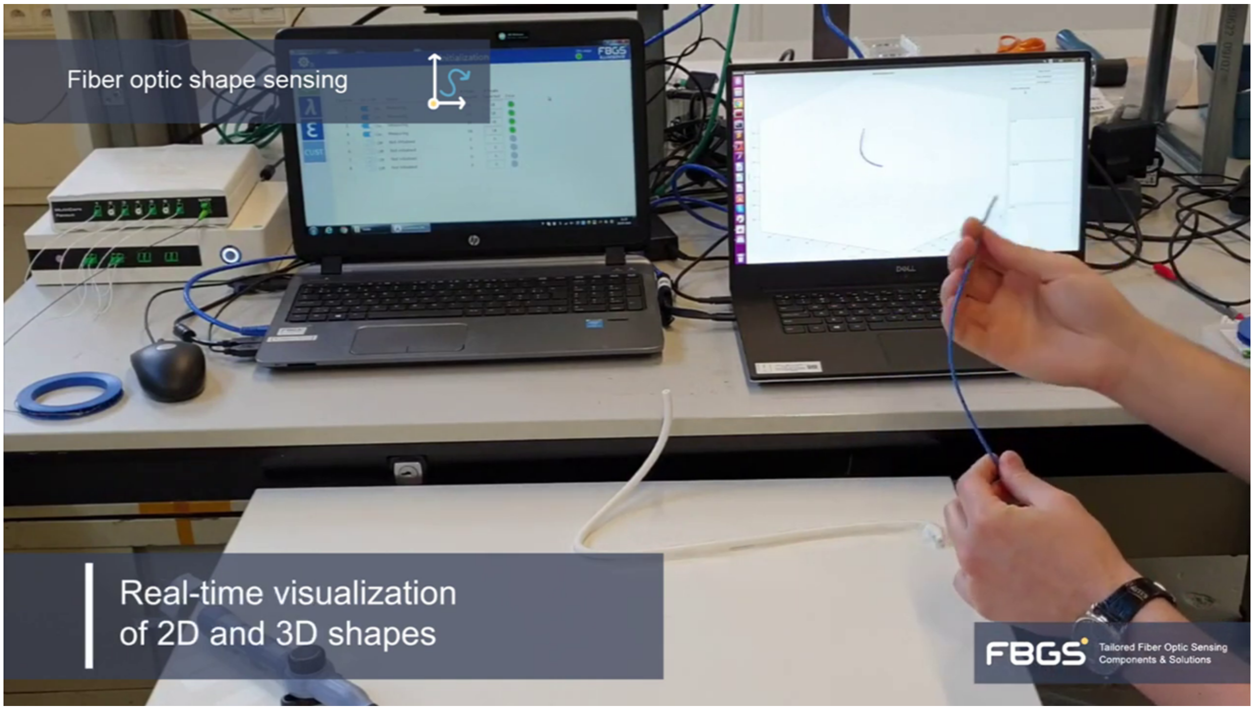

Fig. R18: Real-time shape-sensing optical fibers for medical applications based on the solution offered by the FBGS. Image is taken from YouTube video (https://www.youtube.com/watch?v=U1DgOWDYuiA)

We think a major advantage of MaSoChains is flexibility in design and customization. The purpose of Fig. 6 is to demonstrate that MaSoChain can function as a powerful platform for integrating off-the-shelf electronics, not to show that we have already developed a shape-sensing catheter that has superior performance compared to existing technologies. It is still too early to fully evaluate the potential for MaSoChain in clinical applications, and we are looking forward to developing it together with the community.

4) Although it is claimed that the orientation of the locking magnets has been chosen in such a way to cancel the overall torques in external homogeneous magnetic field, it is obvious that no perfect parallel alignment of the magnets can be achieved due to e.g. the nonideal rigidity of the magnet holding structures, 3D printing tolerances and actual magnetization process of the magnets. Du to these sources of misalignment it is impossible to achieve a full compensation of the overall moment for the pair of magnets. Could you please provide estimates and analysis on how these tolerances affect the shape recovery process, locking and actuation in external homogeneous field for at least three orthogonal field orientations with respect to the structure? Please expand the discussion on this matter.

We completely agree with the reviewer that the alignment of the assembly magnets is never perfect. In fact, based on our experiences, we estimate that the assembly error in terms of the angle of each magnet is approximately 5 degrees. The resulting nonzero dipole moment will lead to an unwanted torque under the external magnetic field (e.g., in Fig. 5). Luckily, this magnetic torque due to the assembly error can be easily estimated as:

$$m_{error} = m_1 \sin\theta = vM_{NdFeB} \sin\theta$$

$$\tau_{error} = m_{error} \times B$$

where m_{error} is the dipole moment due to the alignment error for a single assembly magnet (1.2x1x0.5 mm), M_{NdFeB} is the magnetization of NdFeB (1.08x10^6 A/m) and B is the magnetic flux of the external magnetic field. If we assume that the misalignment angle is 5 degrees and the external magnetic field flux is 20 mT, the corresponding maximum magnetic torque can be calculated as$\tau_{error} = 1.16 \times 10^{-6}\ N \cdot m$

If we compare this torque value for the elastic/magnetic torque value in the assembly process ($\sim 10^{-3}\ N \cdot m$ as in Fig. 2), the misalignment torque is three orders of magnitude smaller. The misalignment dipole moment ($5.8 \times 10^{-5} A \cdot m^2$) from the assembly magnet is two orders of magnitude smaller than the dipole moment of the actuation magnet in Fig. 5 ($4.32 \times 10^{-3} A \cdot m^2$).

We think the error due to the misalignment is not significant compared to the other factors (e.g., magnetic field gradient, surface tension between the water–air interface, disturbances and unexpected load during the assembly process). The influence of this assembly error can be easily estimated. In applications, this error can be compensated for by modifying the magnetic field direction, which is usually easy for an external magnetic field system.

5) There is a certain level of concern about the biocompatibility of the applied materials. Please expand the text addressing the biocompatibility aspects.

Thank you for this question. Biocompatibility is crucial for future applications of MaSoChains. We are very happy to expand our considerations on this aspect. Although the 3D-printed prototypes demonstrated in this paper are not completely biocompatible, we believe there are no fundamental limitations to prevent future MaSoChain prototypes from being biocompatible. We will discuss the biocompatibility of materials composed of MaSoChain separately as follows:

Soft segments: Currently used 3D-printed soft material (Agilus, Stratasys) is not biocompatible. However, we envision other biocompatible polymer materials that have similar or better mechanical performances (silicone, polyethylene, polypropylene, polyurethane, polycarbonate, polyetherimide, Pebax, Nylons, etc.) developed using existing technologies.

Rigid segments: Currently used 3D printed rigid material (VeroWhite, Stratasys) is not biocompatible. However, there are biocompatible 3D-printed biocompatible materials (Biocompatible Digital ABS) offered by Stratasys using the same printer. Of course, other biocompatible materials can also be considered depending on the fabrication processes.

NdFeB magnets: NdFeB is not biocompatible, but the small magnets we purchased from HKCM.de have coatings. Specifically, they offer a Parylene coating, which is nontoxic, fungus and bacteria tight, biocompatible, physiologically and toxicologically harmless, and anti-allergic. (more details: https://www.hkcm.de)

Surface coating is also an important method to isolate nonbiocompatible materials and create a well-defined interface with complex in vivo environments. For envisioned future applications, we believe MaSoChain-based medical devices will be outsourced to qualified companies (e.g., Interplex) for customization and certification before clinical trials.

6) Please add scalebars in Fig. 1, 4, 6, 7.

Thank you for the suggestion. We add them accordingly.

References:

1. Calzolari, V. *et al.* Ablation catheter orientation: In vitro effects on lesion size and in vivo analysis during PVI for atrial fibrillation. *Pacing Clin. Electrophysiol.* **43**, 1554–1563 (2020).
2. Wittkampf, F. H. M. & Nakagawa, H. RF catheter ablation: lessons on lesions. *Pacing Clin. Electrophysiol.* **29**, 1285–1297 (2006).
3. Peyron, Q. *et al.* Kinematic analysis of magnetic continuum robots using continuation method and bifurcation analysis. *IEEE Robot. Autom. Lett.* (2018) doi:10.1109/LRA.2018.2855803.
4. Miyashita, S. *et al.* Ingestible, controllable, and degradable origami robot for patching stomach wounds. in *2016 IEEE international conference on robotics and automation (ICRA)* 909–916 (2016).
5. Bellinger, A. M. *et al.* Oral, ultra--long-lasting drug delivery: Application toward malaria elimination goals. *Sci. Transl. Med.* **8**, 365ra157--365ra157 (2016).
6. Martin, J. W. *et al.* Enabling the future of colonoscopy with intelligent and autonomous magnetic manipulation. *Nat. Mach. Intell.* **2**, 595–606 (2020).
7. Barbot, A., Tan, H., Power, M., Seichepine, F. & Yang, G.-Z. Floating magnetic microrobots for fiber functionalization. *Sci. Robot.* **4**, eaax8336 (2019).
8. Han, M. *et al.* Catheter-integrated soft multilayer electronic arrays for multiplexed sensing and actuation during cardiac surgery. *Nat. Biomed. Eng.* **4**, 997–1009 (2020).
9. Liu, Z. *et al.* Creating three-dimensional magnetic functional microdevices via molding-integrated direct laser writing. *Nat. Commun.* **13**, 1–11 (2022).
10. Alcântara, C. C. J. *et al.* Mechanically interlocked 3D multi-material micromachines. *Nat. Commun.* **11**, 1–8 (2020).
11. Zhang, S. *et al.* On-skin ultrathin and stretchable multifunctional sensor for smart healthcare wearables. *npj Flex. Electron.* **6**, 1–12 (2022).

REVIEWERS' COMMENTS

Reviewer #2 (Remarks to the Author):

The paper has been adequately revised and now the authors have demonstrated to be well aware of the limitations and potential of their solution. The additional simulations and tests, as well as their additional considerations and insights have contributed substantially to my final positive evaluation.

Typos: E.g. Fig 4a, mangent instead of magnet.

Reviewer #3 (Remarks to the Author):

I thank the Authors for their detailed answer. There are some recent works by B. Rivkin et al. (10.1002/aisy.202000238 and 10.1126/sciadv.abl5408) to which the Authors may want to draw their attention while discussing microscale thetered systems, shape and position sensing. Otherwise my questions have been addressed and the manuscript can be considered for publication.

Reviewer #2 (Remarks to the Author):

The paper has been adequately revised and now the authors have demonstrated to be well aware of the limitations and potential of their solution. The additional simulations and tests, as well as their additional considerations and insights have contributed substantially to my final positive evaluation.

Typos: E.g. Fig 4a, mangent instead of magnet.

Thank you for the support and positive evaluation of our work, and we correct the typo in Fig.4

Reviewer #3 (Remarks to the Author):

I thank the Authors for their detailed answer. There are some recent works by B. Rivkin et al. (10.1002/aisy.202000238 and 10.1126/sciadv.abl5408) to which the Authors may want to draw their attention while discussing microscale thetered systems, shape and position sensing. Otherwise my questions have been addressed and the manuscript can be considered for publication.

Thank you for the wonderful suggestions. Indeed, those works are directly relevant and we added those two papers in the references.
